# Supplementary material for: Encephalopathy-linked UFM1 variants impede neuronal protein translation, development, and function
Source: EMBO Mol Med. 2026 Feb 23;18(4):1265–91. doi: 10.1038/s44321-026-00389-6 (PMC13083916; doi:10.1038/s44321-026-00389-6)
Supplement: Supplementary file 1 — Appendix [file 44321_2026_389_MOESM1_ESM.pdf]

Encephalopathy-linked *UFM1* variants impede neuronal protein translation, development, and function

Catarina Perdigão[1], Josefa Torres[1], Helge M. Magnussen[2], Janina Koch[3], Elena Rudashevskaya[1], Frederieke Moschref[1], Maksims Fiosins[4], Fritz Benseler[1], Sally Wenger[1], Tanja Nilsson[1], Sabine Beuermann[1], Stefan Bonn[4, 5], Silvio O. Rizzoli[6], Yogesh Kulathu[2], Olaf Jahn[1,7], Benjamin H. Cooper[1], Mateusz C. Ambrozkiewicz[3], JeongSeop Rhee[1], Nils Brose[1*], Marilyn Tirard[1*]

[1] Max Planck Institute for Multidisciplinary Sciences, Department of Molecular Neurobiology, Göttingen, Germany

[2] MRC Protein Phosphorylation and Ubiquitylation Unit, School of Life Sciences, University of Dundee, Dundee, UK

[3] Institute of Cell Biology and Neurobiology, Charité-Universitätsmedizin Berlin, Berlin, Germany

[4] Institute of Medical Systems Bioinformatics, Centers for Biomedical AI (bAIome), Center for Molecular Neurobiology (ZMNH) and Translational Immunology (HCTI), University Medical Center Hamburg-Eppendorf, Hamburg, Germany

[5] German Center for Child and Adolescent Health (DZKJ), Partner Site Hamburg, University Medical Center Hamburg-Eppendorf, Germany

[6] Department of Neuro- and Sensory Physiology, University Medical Center Göttingen, Göttingen, Germany

[7] Department of Psychiatry and Psychotherapy, University Medical Center Göttingen, Göttingen, Germany

* Co-corresponding authors

Table of Contents

Appendix Figure S1: Characterization of the CRISPR/Cas9-mediated depletion of UFM1 used for IUE. 3

Appendix Figure S2: UFM1 depletion reduces neuronal complexity *in vitro* early during development. 4

Appendix Figure S3: UFM1 depletion does not change the localization and intensity of pre- and postsynaptic proteins. 6

Appendix Figure S4: UFM1 depletion alters inhibitory synaptic transmission. 7

Appendix Figure S5: Comparative 3D ultrastructural analysis of UFM1-deficient (CRE) and littermate control (RFP) synapses. 10

Appendix Figure S6: RNA-seq analysis of UFM1-KO and RFP control cells. 13

Appendix Figure S7: Gene ontology of down-regulated genes upon UFM1 depletion. 14

Appendix Figure S8: Gene ontology for up-regulated genes upon UFM1 depletion. 15

Appendix Figure S9: Expression of the exogenous *UFM1* variants 16

Appendix Figure S10: Characterization of exogenous HA-UFM1 mutant expression in HEK cells by LC-MS/MS. 18

Appendix Figure S11: Characterization of UFM1 mutant expression in primary neurons by LC-MS/MS. 20

Appendix Figure S12: Neuronal expression of UFM1-R81C does not fully rescue neuronal complexity *in vitro*. 22

Appendix Figure S13: Variation in mEPSC amplitudes during autaptic culture maturation 23

Appendix Figure S14: Characterization of the UPR response. 24

Appendix Figure S15: Neuronal expression of UFM1-R81C does not change the ER membrane fluidity 26

Appendix Figure S16: Trazodone treatment does not alter neuronal morphology 27

Appendix Figure S17: Identification of mouse brain UFM1 candidate targets 28

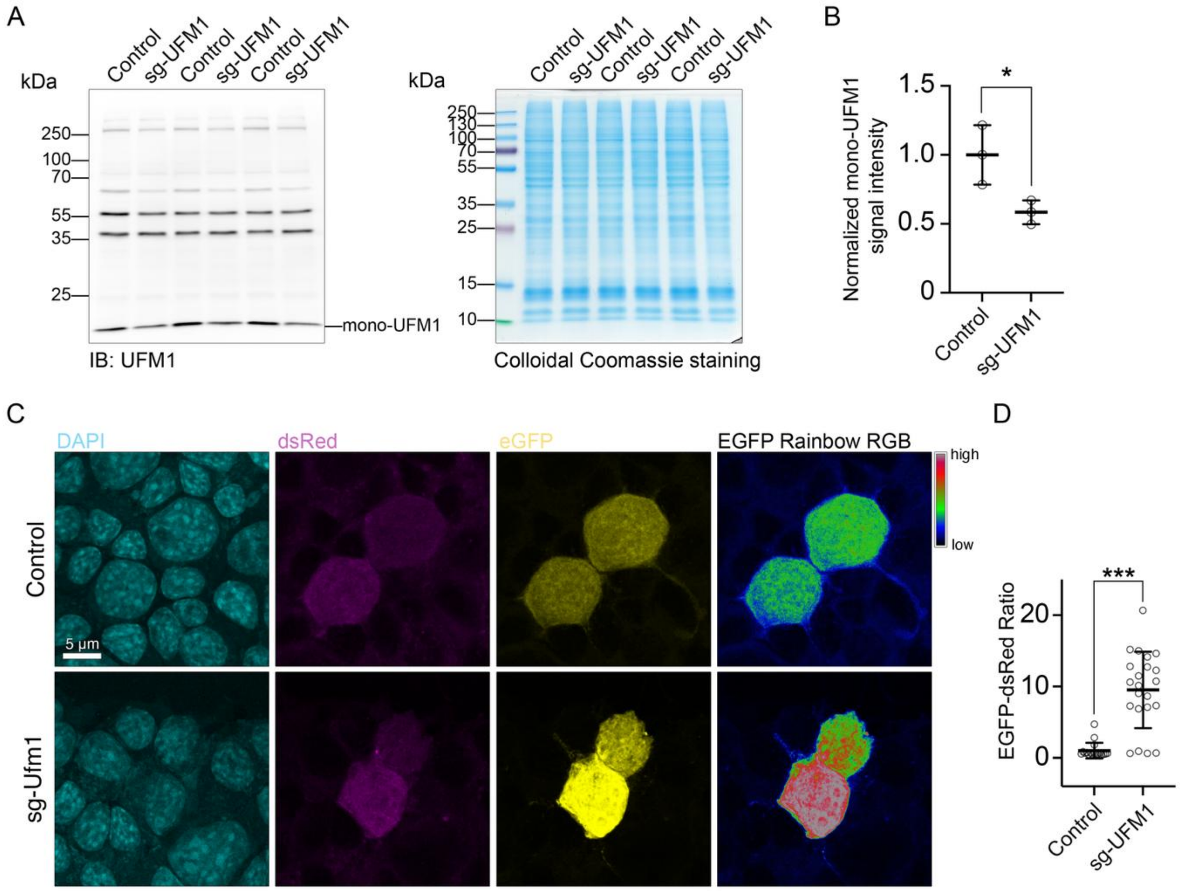

Appendix Figure S1: Characterization of the CRISPR/Cas9-mediated depletion of UFM1 used for IUE.

(A) Anti-UFM1 immunoblot (IB: UFM1) of neuroblastoma N2a cell lysates transfected either with control plasmid (Control) or bicistronic vector for the expression of Cas9 and sg-RNA to target UFM1 gene (sg-UFM1). Colloidal Coomassie staining (CC) was used to visualize all proteins resolved in the gel and normalized mono-UFM1 levels in B.

(B) Bar graph showing the normalized mono-UFM1 level in the experiment in (A). mono-UFM1 level were normalized to the total protein per lane and expressed relative to the control.

(C) Validation of the CRISPR editing efficiency of the sg-UFM1. Neuroblastoma N2a cells were co-transfected with pEGxxFP plasmid containing the DNA sequence targeted by sg-UFM1 and dsRed expression vector.

(D) Quantification of the editing efficiency as the ratio of EGFP-to-dsRed fluorescence per cell.

For B and D, horizontal line depicts the mean and the error bars, ± S.D. A Shapiro-Wilk normality test and two-tailed unpaired t-test (B), or Mann Whitney test (D) were used. *** , $p < 0.001$; * , $p < 0.05$.

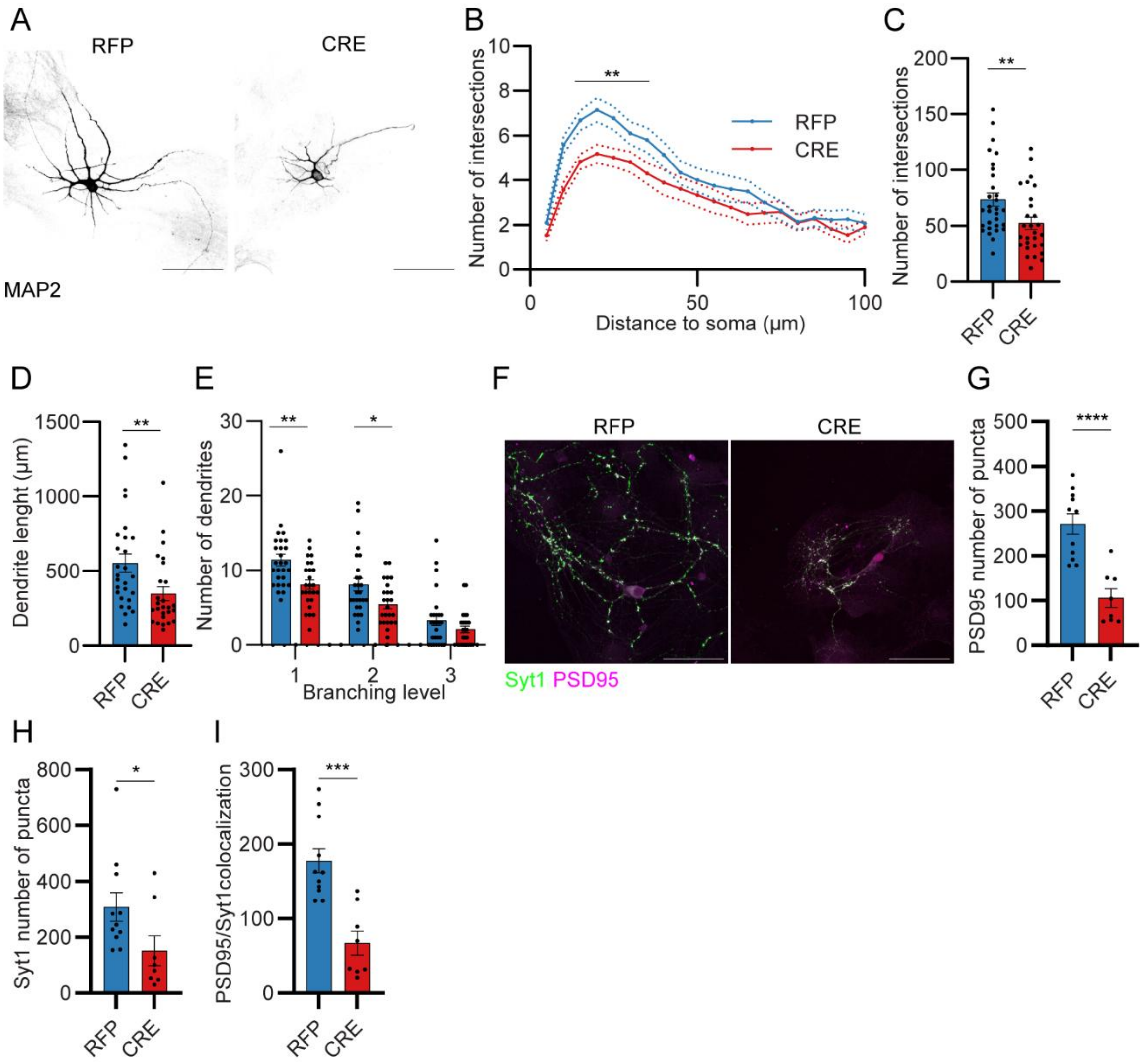

Appendix Figure S2: UFM1 depletion reduces neuronal complexity *in vitro* early during development.

(A) Representative images of primary hippocampal UFM1-cKO neurons infected at DIV 1 with RFP or CRE expressing viruses, fixed and immunolabelled for MAP2 (black) at DIV 5. Scale bar, 50 μm.

(B) Sholl analysis line graph depicting the average number of dendrite intersections with concentric circles radiating every 5 μm from the soma, using traces as shown in (A).

(C) Bar graph showing the total number of dendrite intersections, obtained from the Sholl analysis in (B).

(D) Bar graph showing the dendrite lengths, obtained from traces in (A).

(E) Bar graph showing the number of primary, secondary and tertiary branching from dendrites (branching levels 1, 2 or 3, respectively) of neurons as shown in (A).

Data of B-E were obtained from N = 3 independent experiments, n = 27 RFP and 26 CRE neurons. **, $p<0.01$; *, $p<0.05$ using a D'Agostino-Pearson normality test and Mann Whitney test. Dotted lines and bar graphs show mean ± SEM.

(F) Representative images of primary hippocampal UFM1-cKO neurons, infected at DIV 1 with RFP (left panel) or CRE (right panel) expressing viruses, fixed and immunolabelled for Synaptotagmin1 (green) and PSD95 (magenta) at DIV 13. Scale bar, 50 µm.

(G-I) Bar graphs depicting the total number of PSD95 (G), Synaptotagmin1 (H) and PSD95/Synaptotagmin1 co-localized puncta (I), obtained from neurons as shown in (F).

Data of G-I were obtained from N = 2 independent experiments, n = 11 RFP and 8 CRE neurons. ****, $p<0.0001$; ***, $p<0.001$; *, $p<0.05$ using D'Agostino-Pearson normality test and Unpaired t-test (G, I) or Mann-Whitney test (H). Bar graphs show mean ± SEM.

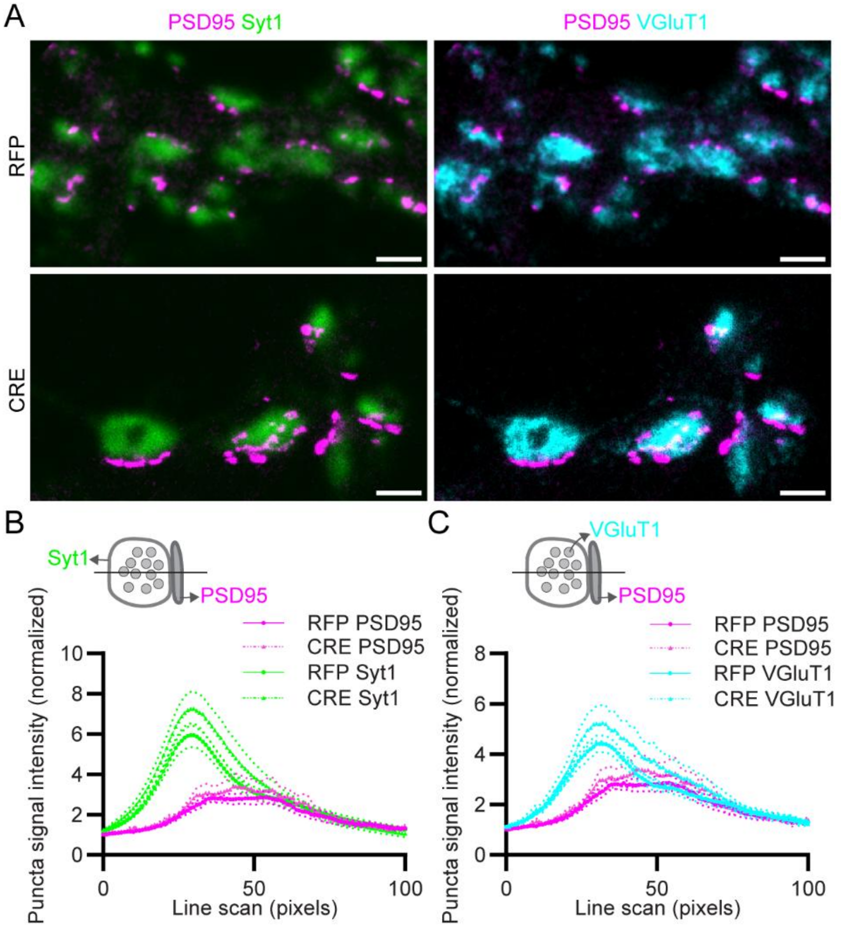

Appendix Figure S3: UFM1 depletion does not change the localization and intensity of pre- and postsynaptic proteins.

(A) Representative STED images of primary hippocampal UFM1-cKO neurons, infected at DIV 1 with RFP (top panels) or CRE (bottom panels) expressing viruses, fixed and immunolabelled for Synaptotagmin1 (Syt1, green), PSD95 (magenta), and VGluT1 (cyan) at DIV 13. Scale bar, 1 µm.

(B) Line graph depicting the intensity profile of Syt1 and PSD95 along a 100 pixels line scan, from images in (A).

(C) Line graph depicting the intensity profile of VGluT1 and PSD95 along a 100 pixels line scan, from images in (A).

Data of B-C from N=3 independent experiments, n= 9 RFP and 9 CRE cells. For (B), p=0.6905 using a Mann Whitney test. Line graphs and dots show mean ± SEM

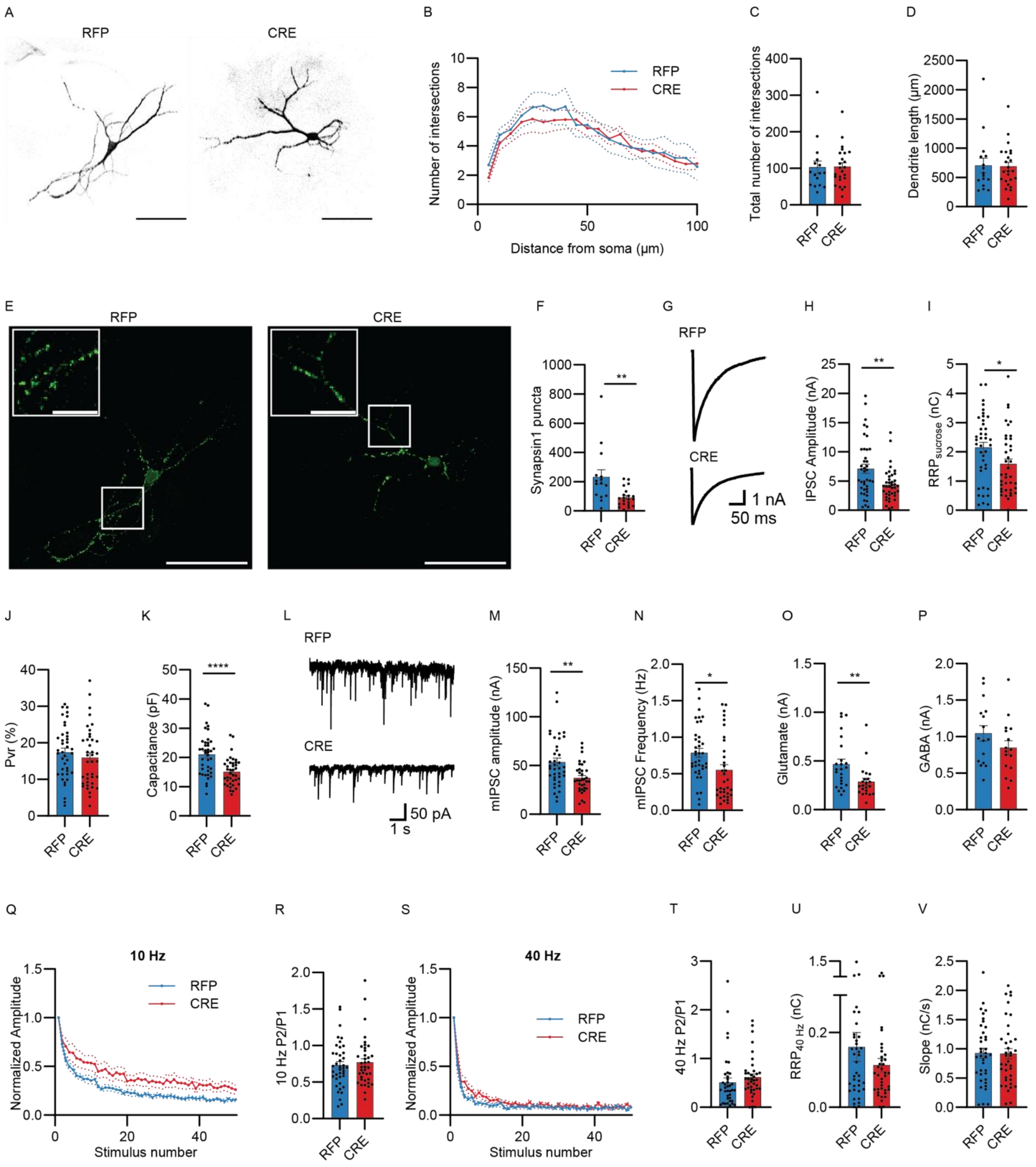

Appendix Figure S4: UFM1 depletion alters inhibitory synaptic transmission.

(A) Representative images of primary striatal UFM-cKO neurons, infected at DIV 1 with RFP (left panel) or CRE (right panel) expressing viruses, fixed and immunolabelled for MAP2 (black) at DIV 13. Scale bar, 50 μm.

(B) Sholl analysis line graph depicting the average number of intersections between dendrites and concentric circles radiating every 5 μm from the soma, using neuronal traces as shown in A.

(C) Bar graph showing the total number of dendrite intersections, obtained from the Sholl analysis in B.

(D) Bar graph showing the dendrite lengths, obtained from traces in A.

(E) Representative images of primary striatal UFM1-cKO neurons, infected at DIV 1 with RFP (left panel) or CRE (right panel) expressing viruses, fixed and immunolabelled at DIV 13 for Synapsin1 (green). Scale bar, 50 μm, white frame inset 10 μm.

(F) Bar graph depicting the total number of Synapsin1 puncta, obtained from neurons as shown in E.

(G) Representative current traces of evoked inhibitory postsynaptic currents (IPSC) of DIV 10-12 primary UFM1-cKO striatal autaptic neurons infected at DIV 1 with RFP (up) and CRE (bottom) expressing viruses.

(H-K) Bar graphs depicting IPSC amplitude (H), the charge transferred by the release of the readily-releasable pool of synaptic vesicles upon treatment with a hypertonic sucrose solution ($RRP_{sucrose}$) (I), the vesicular release probability (P_{vr}) (J), and the membrane capacitance (K).

(L) Representative traces of spontaneous miniature IPSC (mIPSC) of DIV 10-12 autaptic UFM1-cKO neurons infected at DIV 1 with RFP (up) and CRE (bottom) expressing viruses.

(M, N) Bar graphs depicting mIPSC amplitude (M) and frequency (N).

(O, P) Bar graphs depicting the amplitude of the peak current generated by the postsynaptic responses to the application of glutamate (100 μM) (O) or γ-aminobutyric acid (GABA, 3 μM) in DIV 10-12 autaptic UFM1-cKO neurons infected at DIV 1 with RFP and CRE expressing viruses (P).

(Q) Line graph depicting the average normalized IPSC amplitudes during 10 Hz stimulation trains.

(R) Bar graph showing paired-pulse ratios (P2/P1) obtained from Q. The amplitude of the second stimulus was normalized by the amplitude of the first one.

(S) Line graph depicting the average normalized IPSC amplitudes during 40 Hz stimulation trains.

(T) Bar graph showing paired-pulse ratios (P2/P1) obtained from S.

(U) Bar graph showing the size of readily releasable pool (RRP_{40Hz}), estimated by back extrapolation of the cumulative IPSC after 40Hz stimulus trains.

(V) Bar graph showing the rate of vesicle replenishment (slope) estimated by the slope of the back extrapolated cumulative IPSC curve of the 40Hz stimulus trains.

Data of A-D were obtained from N=3 independent experiments with n= 16 RFP and 24 CRE cells. A D'Agostino-Pearson normality test and a Mann Whitney test were used. Dotted lines and bar graphs show mean ± SEM.

Data of E-F were obtained from N=3 independent experiments with n= 15 RFP and 21 CRE cells. **$p<0.01$ using a D'Agostino-Pearson normality test and a Mann Whitney test. Bar graph shows mean ± SEM.

Data of G-K were obtained from N=5 independent experiments with n = 40 RFP and 39 CRE neurons. *, $p<0.05$; **, $p<0.01$; ****, $p<0.0001$ using a D'Agostino-Pearson normality test, a Mann Whitney test, or an unpaired t-test. Bar graphs show mean ± SEM.

Data of L-N were obtained from N=5 independent experiments with n = 38 RFP and 34 CRE neurons. *$p<0.05$, **$p<0.01$ using a D'Agostino-Pearson normality test, a Mann Whitney test, or an unpaired t-test. Bar graphs show mean ± SEM.

Data of O were obtained from N=5 independent experiments with n = 24 RFP and 22 CRE neurons. **$p<0.01$ using a D'Agostino-Pearson normality test and a Mann Whitney test. Bar graph shows mean ± SEM.

Data of P were obtained from N=5 independent experiments with n = 16 RFP and 16 CRE neurons. A D'Agostino-Pearson normality test and an unpaired t-test were used. Bar graph show mean ± SEM.

Data of Q-R were obtained from N=5 independent experiments with n= 40 RFP and 39 CRE cells. A D'Agostino-Pearson normality test and a Mann Whitney test were used. Dotted lines and bar graphs show mean ± SEM.

Data of S-V were obtained from N=5 independent experiments with n= 38 RFP and 38 CRE cells. A D'Agostino-Pearson normality test, a Mann Whitney test, or an unpaired t-test were used. Dotted lines and bar graphs show mean ± SEM.

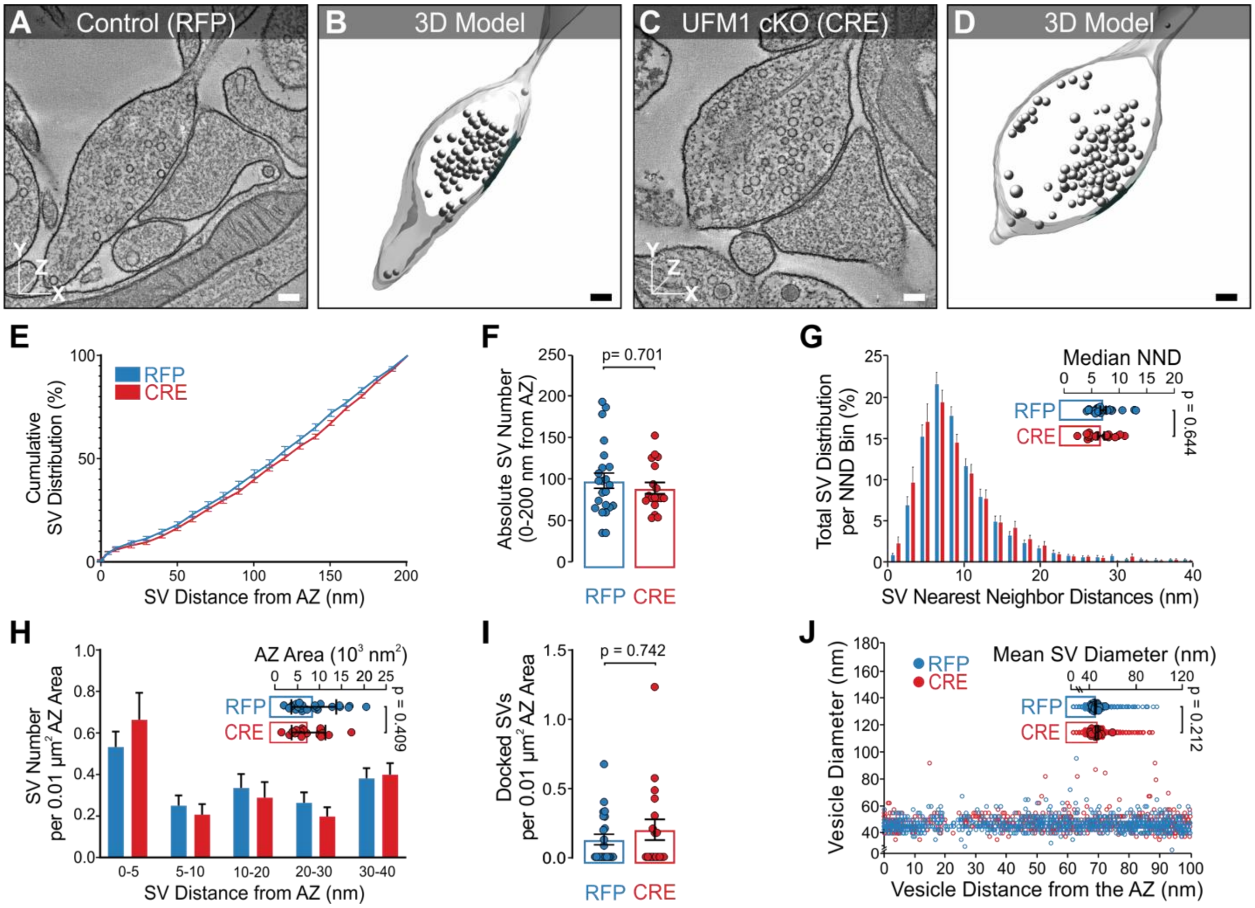

Appendix Figure S5: Comparative 3D ultrastructural analysis of UFM1-deficient (CRE) and littermate control (RFP) synapses.

(A-D) Tomographic slices through representative control (A, RFP) and UFM1-deficient (C, CRE) presynaptic boutons. B and D, with corresponding 3D models. Scale bar: 100 nm.

(E) Cumulative plot depicting the relative SV distribution within 0-200nm of the AZ.

(F) Scatter plot indicating total SV number quantified within 0-200 nm of the AZ. p=0.701 via Mann-Whitney test.

(G) Histogram showing the distribution of inter-SV nearest-neighbor distances (NND) quantified from all reconstructed SVs (bin size=2 nm). NND values exceeding the 0-40 nm range were not plotted. Inset graph depicts median NND values. p=0.644 with Welch's t-test.

(H) Histogram showing the spatial distribution of SVs within 0-40 nm of the AZ. Values were normalized to corresponding AZ areas. Inset graph depicts AZ areas quantified for the two conditions. p=0.3927 with Welch's test.

(I) Scatter plot indicating the number of docked SVs normalized to AZ area. p=0.7417 via Mann-Whitney test.

(J) Histogram showing the distribution of inter-vesicular nearest-neighbor distances (NND) for SVs within 0-40 nm of the AZ. Inset graph depicts median NND values quantified for the two conditions. p=0.2122 with Mann-Whitney test.

N=3 independent experiment with n=23 tomograms for RFP (control) and n=18 tomograms for CRE (UFM1 deficient).

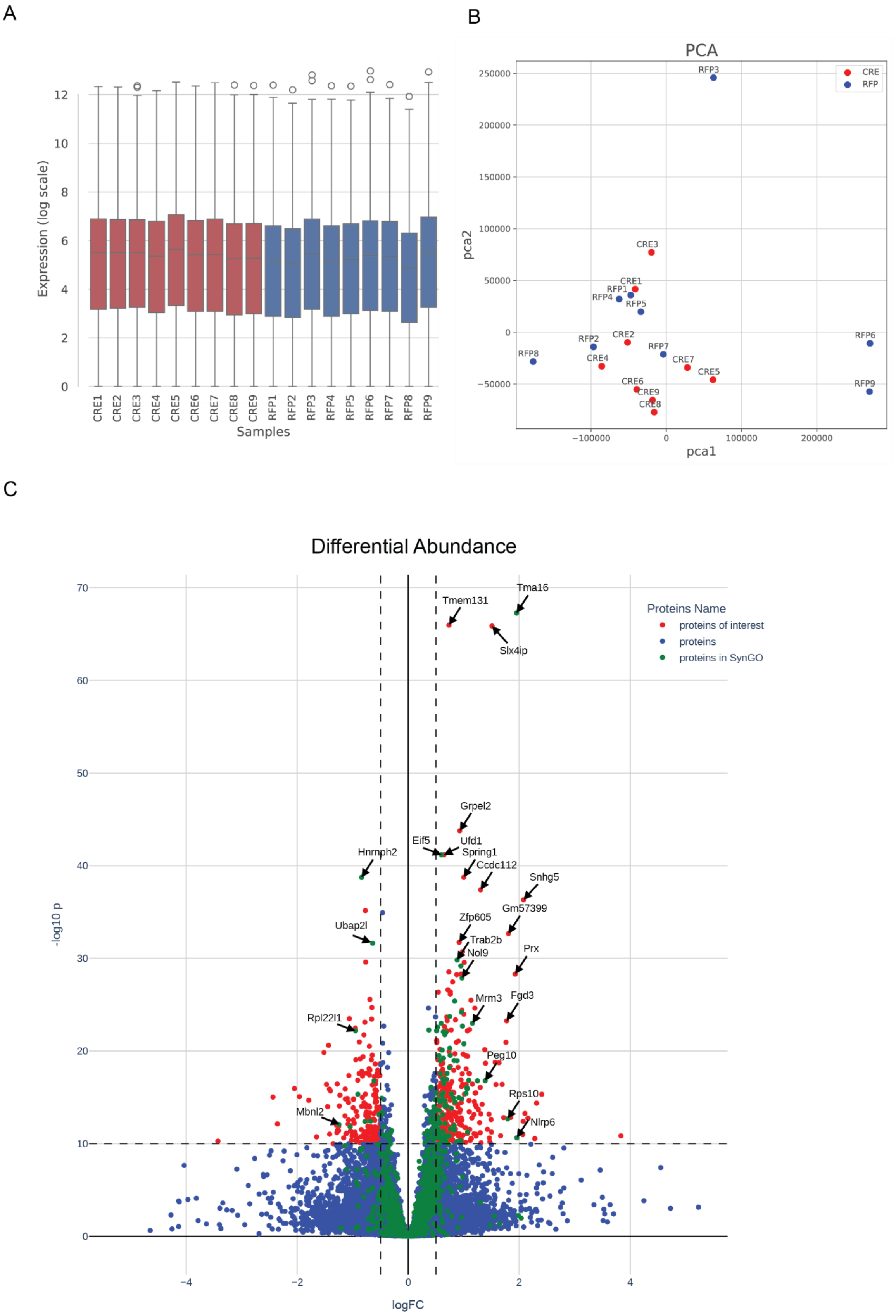
A
B
C
Expression (log scale)
Samples
CRE1
CRE2
CRE3
CRE4
CRE5
CRE6
CRE7
CRE8
CRE9
RFP1
RFP2
RFP3
RFP4
RFP5
RFP6
RFP7
RFP8
RFP9
PCA
pca1
pca2
CRE
RFP
Differential Abundance
-log10 p
logFC
Proteins Name
proteins of interest
proteins
proteins in SynGO
Tma16
Tmem131
Slx4ip
Grpel2
Eif5
Ufd1
Spring1
Hnrnph2
Ccdc112
Snhg5
Gm57399
Zfp605
Ubap2l
Trab2b
Nol9
Prx
Mrm3
Fgd3
Rpl22l1
Peg10
Rps10
Mbnl2
Nlrp6

Appendix Figure S6: RNA-seq analysis of UFM1-KO and RFP control cells.

(A, B) Total gene counts (A) and PCA (B) analysis verifying the quality of the samples and of the analysis.

(C) Volcano plot visualization of changes in gene expression upon deletion of UFM1 in primary hippocampal neurons. RNA was extracted from DIV 13 UFM1cKO primary hippocampal neurons infected at DIV 1 with lentivirus expressing RFP as control, or CRE-recombinase to deplete UFM1, and sequenced. The volcano plot depicts each gene based on its Log2 fold of change (logFC) and the reverse log10 of their p value (-log10p). Blue dots correspond to genes whose expression was below threshold. Red dots correspond to genes with a logFC above 0.5 and -lop10p above 10, as indicated by the black dotted lines. Green dots correspond to genes found in the SynGO database. Relevant selected genes names are indicated with an arrow.

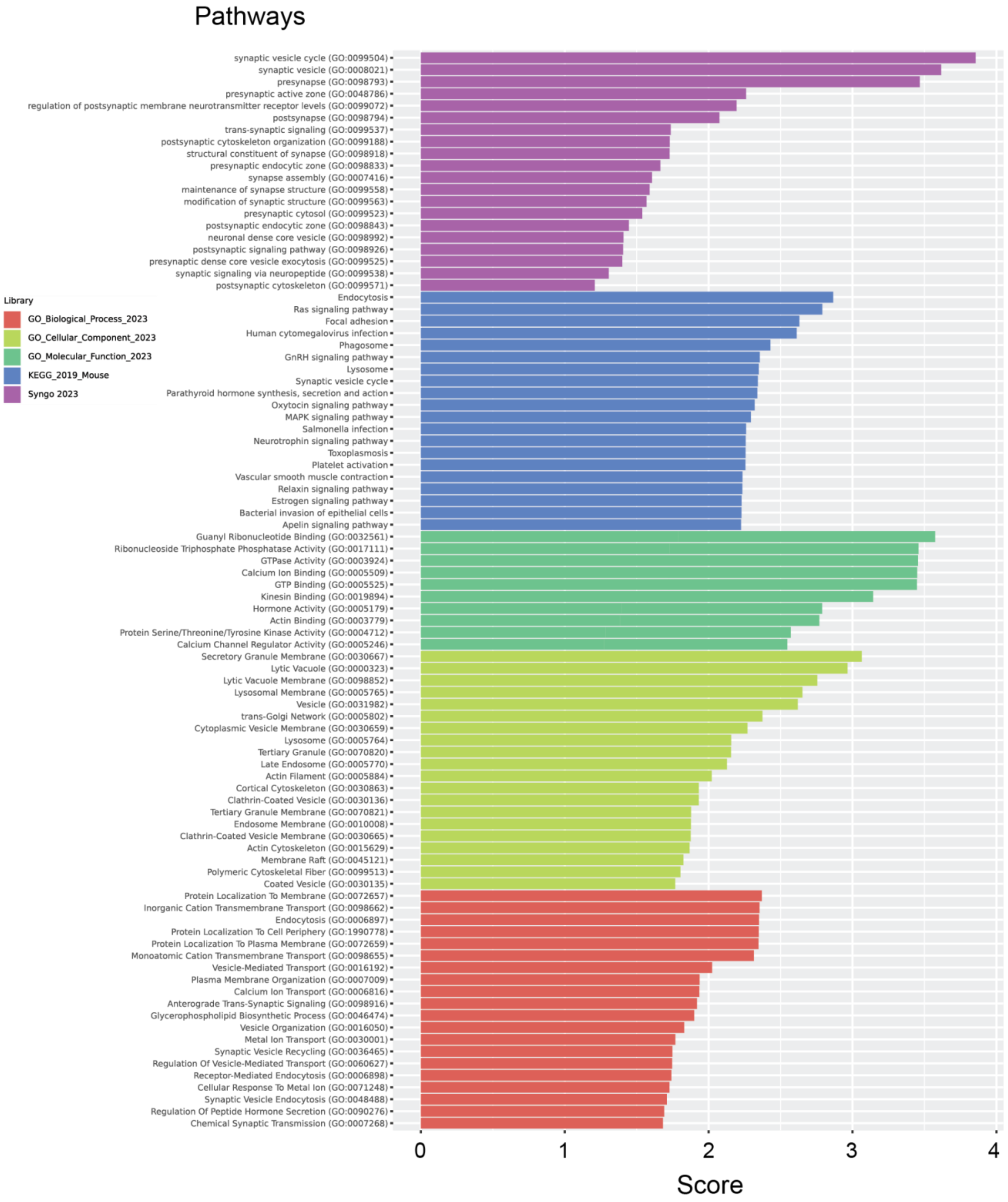

Appendix Figure S7: Gene ontology of down-regulated genes upon UFM1 depletion.

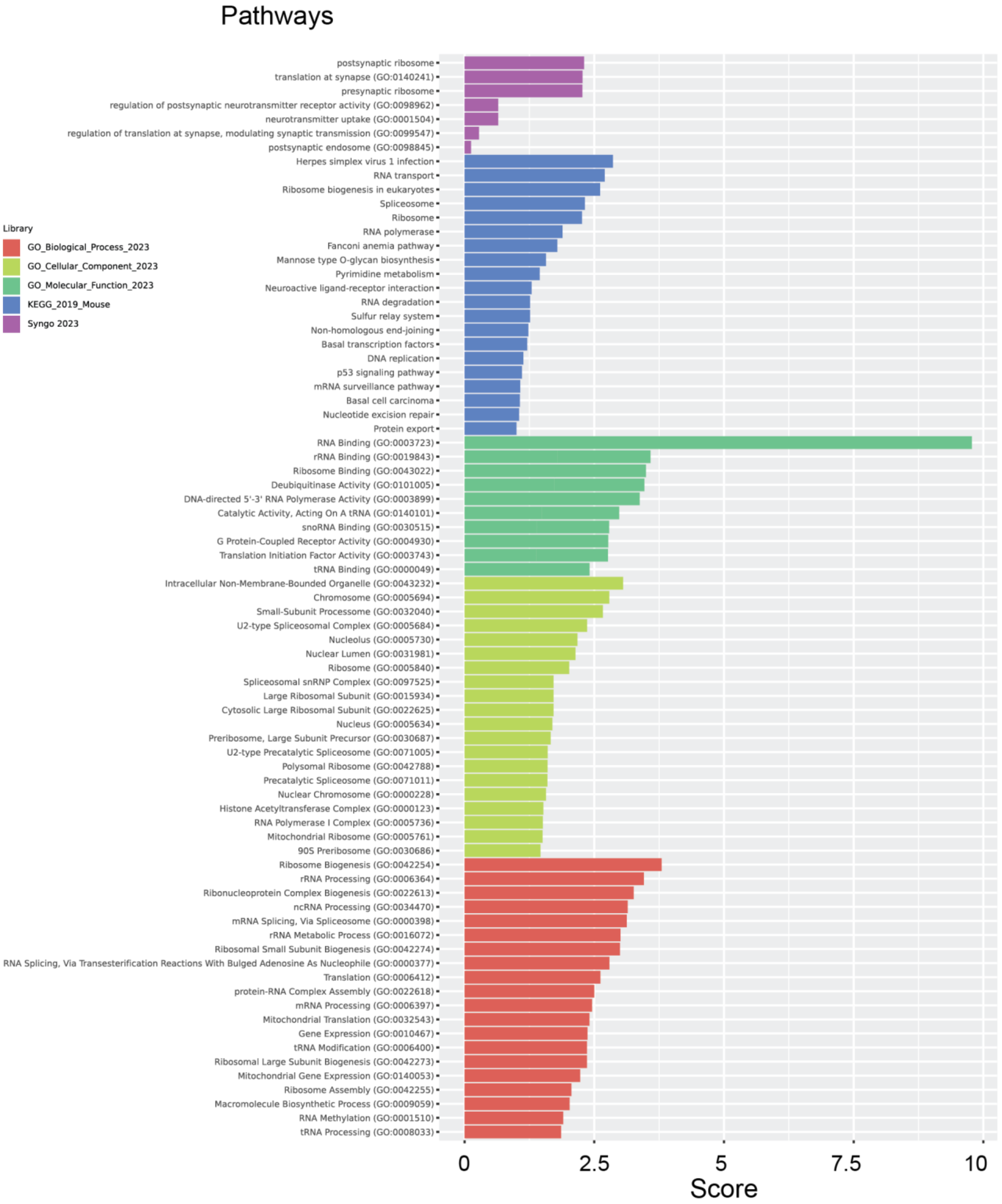

Appendix Figure S8: Gene ontology for up-regulated genes upon UFM1 depletion.

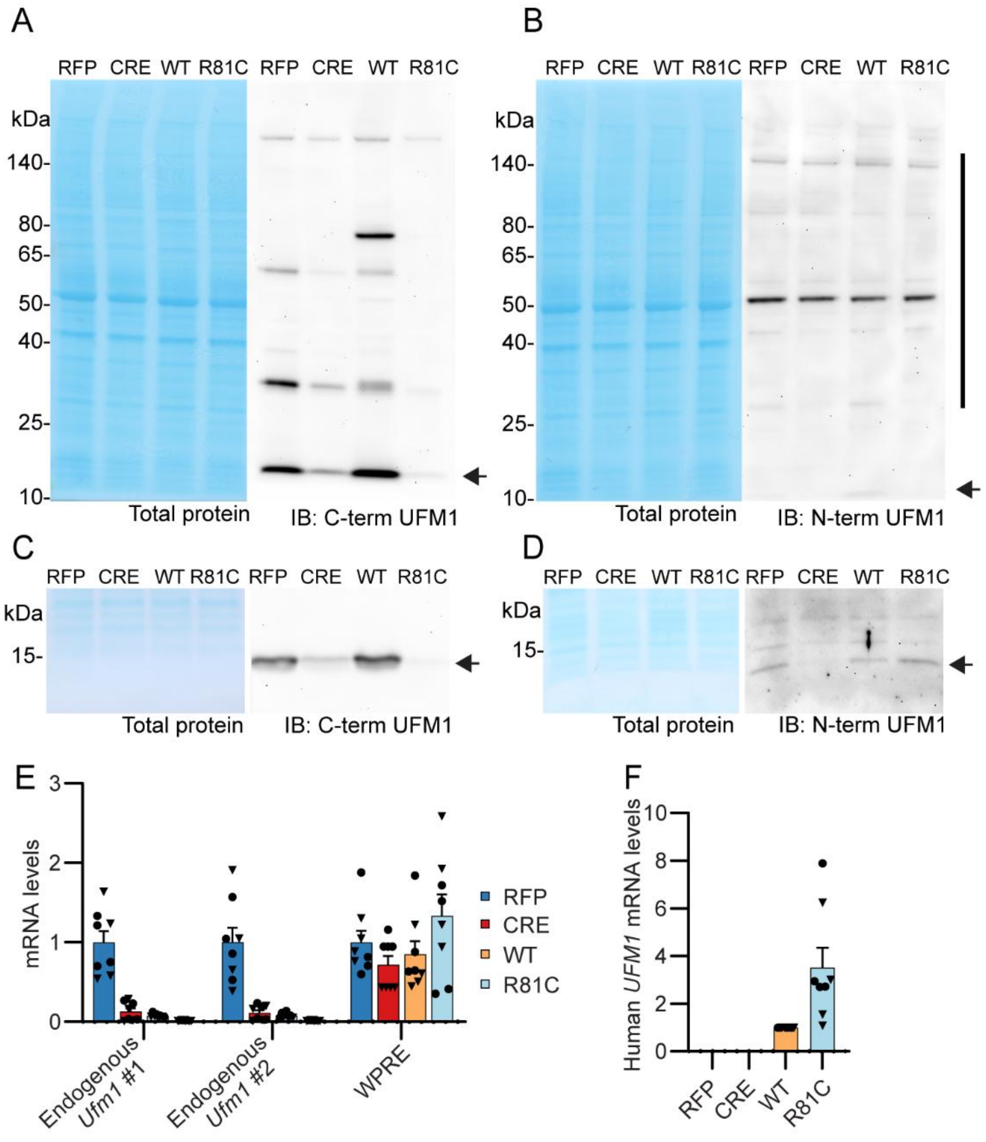

Appendix Figure S9: Expression of the exogenous *UFM1* variants

(A-D) Anti-UFM1 IB analysis of lysates from DIV 10 primary UFM1-cKO hippocampal neurons infected at DIV 1 with viruses expressing RFP, CRE, CRE combined with UFM1-WT (WT) or UFM1-R81C (R81C). Arrow, unconjugated UFM1. Black line, UFM1 conjugates. Molecular weights are indicated on the left (kDa). For panels A and C used an antibody detecting the C-terminal region of UFM1 was used (Abcam, ab109305). For panels B and D, an antibody detecting the N-terminal region of UFM1 was used (Novus, NBP2-94235). Panels A and B depict Western

bots of neuronal lysates using a 4-12% Bis-Tris gel, while panels C and D depict Western bots of neuronal lysates using a 15% Bis-Tris gel.

(E) Bar graph depicting mRNA levels of endogenous *Ufm1* using two different pairs of primers (endogenous *Ufm1* #1 and endogenous *Ufm*1 #2), and of WPRE (a viral sequence), determined by qPCR and normalized to *Gapdh* (circles) or *Ubcc* (triangles). mRNAs were extracted from DIV 13 primary UFM1-cKO primary cultured neurons infected at DIV 1 with viruses expressing RFP, CRE, CRE combined with UFM1-WT (WT) or UFM1-R81C (R81C).

(F) Bar graph depicting mRNA levels of exogenously expressed human *UFM1-WT* or *UFM1-R81C* in DIV 13 primary Ufm1-cKO primary cultured neurons infected at DIV 1 with viruses expressing RFP, CRE, CRE combined with UFM1-WT (WT) or UFM1-R81C (R81C), determined by qPCR and normalized to *Gapdh* (circles) or *Ubcc* (triangles). N=4 independent experiments.

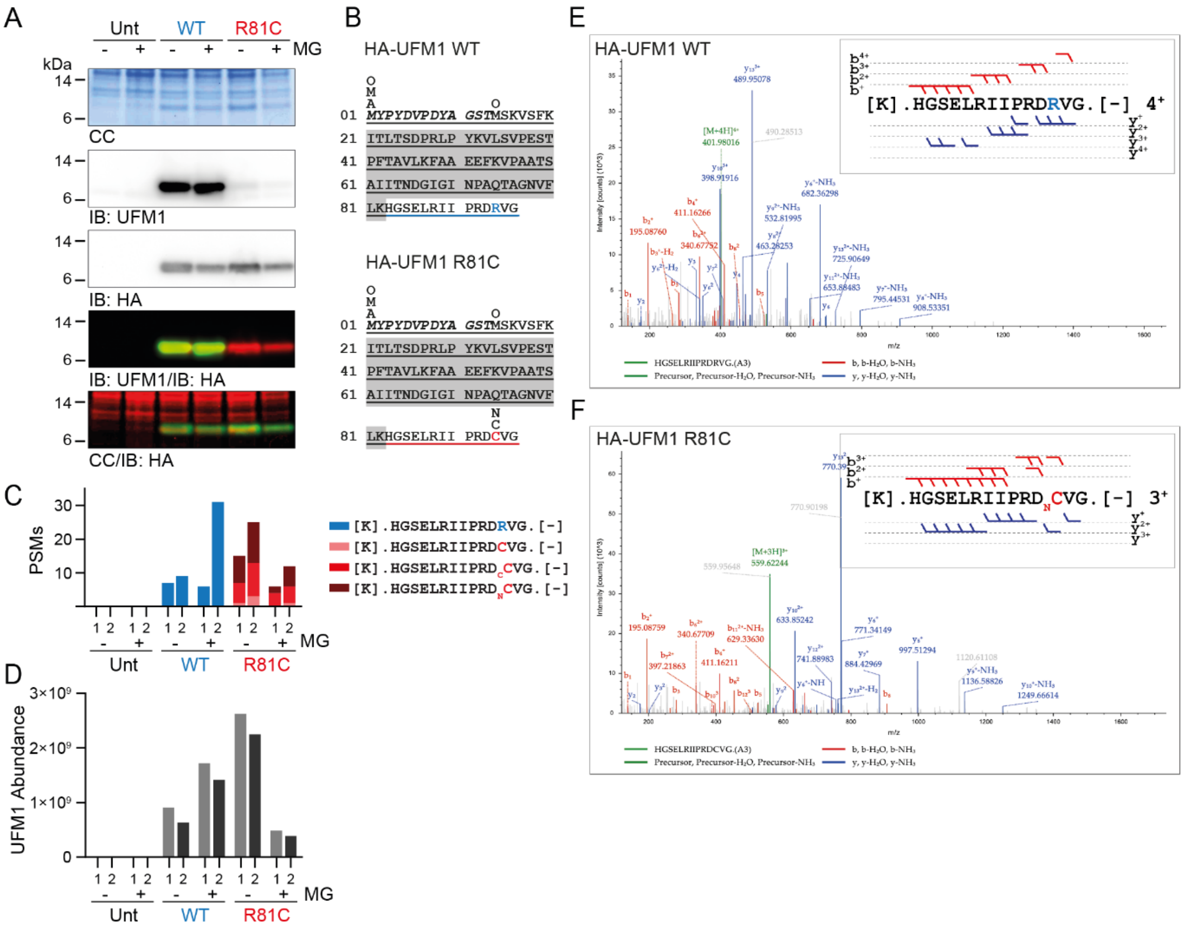

Appendix Figure S10: Characterization of exogenous HA-UFM1 mutant expression in HEK cells by LC-MS/MS.

(A) Lysates from untransfected (Unt) HEK cells or HEK cells overexpressing wild-type (WT) or mutant (R81C) HA-UFM1, treated or not with the proteasome inhibitor MG132 (MG+) or vehicle (DMSO, MG-), were subjected to separation on two parallel SDS-PAGE 4-12% Bis-Tris gels. One gel was used for Colloidal Coomassie staining (CC, top panel) and the other one for immunoblotting with anti-UFM1 (IB:UFM1, second from top panel) and anti-HA (IB: HA, third from top panel) antibodies. The overlay of the immunoblots (IB: UFM1/IB: HA, second from bottom panel) indicates that both antibodies recognize the same protein bands. The overlay of the CC staining and the anti-HA immunoblot (CC/IB: HA, bottom panel) revealed the regions of interest in the Coomassie-stained gel to be excised and subjected to in-gel digestion with endoprotease Lys-C, followed by LC-MS/MS analysis of the cleavage products.

(B) Amino acid sequences of mature HA-UFM1-WT and HA-UFM1-R81C. The presence of HA-UFM1 in both WT and R81C samples was confirmed by 100% protein sequence coverage via mass spectrometric identification of the proteolytic peptides (underlined sequences, HA tag in italic). Identification of the C-terminal peptides of both HA-UFM1 variants with R94 (blue),

corresponding to R81 in untagged UFM1-WT, and HA-UFM1-R81C with the R94C mutation (red), corresponding to R81C in untagged UFM1-R81C, distinguishes HA-UFM1-WT from HA-UFM1-R81C protein, and confirms their expression in corresponding samples as shown in (C). Unmodified peptides shared by both UFM1 variants (highlighted in grey) were used for label-free protein quantification to estimate the abundance of UFM1 as shown in (D). Note that the C-terminal peptide of HA-UFM1-R81C was identified in three forms differing by their cysteine (C94) status, i.e. unmodified, carbamidomethylated or modified by N-ethylmaleimide (marked with C and N above R94C). Similarly, the N-terminal peptide was found in several forms including oxidation (O), loss (M) or acetylation (A) of the N-terminal methionine.

(C) Identification of HA-UFM1-WT (blue) and HA-UFM1-R81C (red) C-terminal peptides. Each sample was analyzed with two different LC-MS/MS methods (1, 2; see Methods section for details). The diagram shows the number of MS/MS scans identified as the respective C-terminal peptide (peptide-spectrum matches, PSMs) in the individual samples. The PSMs for the different Cys-variants of the mutated peptide (see above) were summed up, and indicated with different intensity of the colour.

(D) Comparison of UFM1 abundance in the samples estimated via label-free protein quantification according to the TopN approach. The quantification was based on four most abundant peptides (Top4), unmodified and shared by both UFM1 variants ([K].ITLTSDPRLPYK.[V], [K].VLSVPESTPFTAVLK.[F], [K].FAAEEFK.[V], and [K].VPAATSAIITNDGIGINPAQTAGNVFLK.[H]; highlighted in grey in (B)). Each sample was analyzed with two different LC-MS/MS methods as in (C). Note that HA-UFM1-R81C is expressed at a level comparable to that of HA-UFM1-WT.

(E, F) Fragment ion mass spectra of the C-terminal peptide of HA-UFM1-WT (E, top panel) and HA-UFM1-R81C (N-ethylmaleimide-modified form, F, bottom panel). The respective presence of R94 or C94 (corresponding to R81 and C81 in untagged UFM1 protein variants) was unambiguously confirmed by N- and C-terminal ion series. Only b- (red) and y-ions (blue) are labeled for clarity.

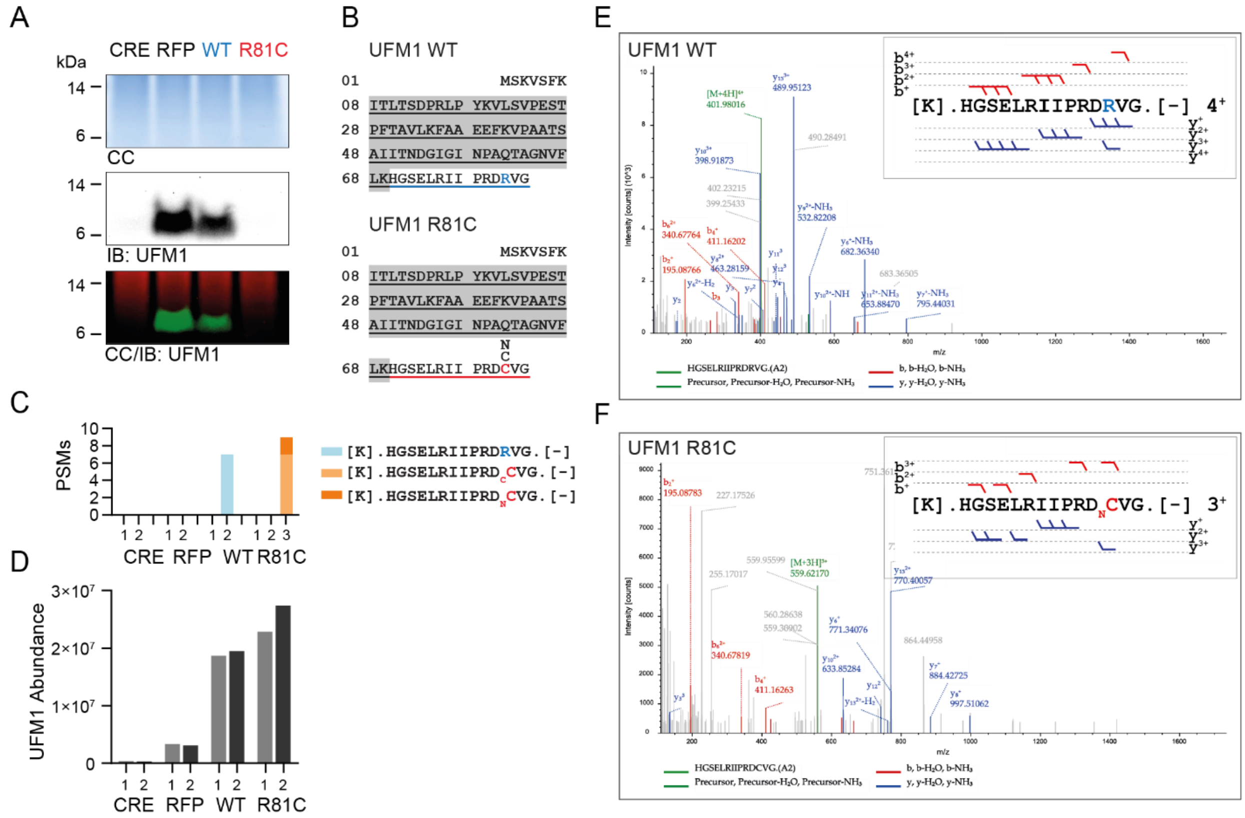

Appendix Figure S11: Characterization of UFM1 mutant expression in primary neurons by LC-MS/MS.

(A) Lysates from primary DIV 12 UFM1-cKO hippocampal neurons infected at DIV 1 with viruses expressing CRE (UFM1 KO), RFP (infection control), and CRE combined with UFM1-WT (WT), or UFM1-R81C (R81C) were subjected to separation on single 4-12% Bis-Tris SDS-PAGE gel with symmetrical sample loading. One half of the gel was used for Colloidal Coomassie staining (CC) and the other for immunoblotting with anti-UFM1 antibodies (IB: UFM1). The overlay of the CC staining and the anti-UFM1 immunoblot (CC/IB: UFM1) revealed the gel regions of interest to be excised and subjected to in-gel digestion with endoprotease Lys-C, followed by LC-MS/MS analysis of the cleavage products.

(B) Amino acid sequences of mature UFM1-WT and UFM1-R81C. The presence of UFM1-WT and UFM1-R81C proteins was confirmed in their respective samples with 91.6% protein sequence coverage by mass spectrometric identification of the proteolytic peptides. Identification of C-terminal peptides of UFM1 protein with R81 (blue) in WT samples and with the R81C mutation (red) in RC samples distinguishes UFM1-WT from UFM1-R81C protein, and confirms their expression in corresponding samples as shown in (C). Unmodified peptides shared by both UFM1 variants (highlighted in grey) were used for label-free protein quantification to estimate the abundance of UFM1 as shown in (D). Note that the C-terminal peptide of UFM1-R81C was

identified in two forms differing by their cysteine status, i.e. carbamidomethylated or modified by N-ethylmaleimide (marked with C and N above R81C).

(C) Identification of C-terminal peptides of UFM1-WT and UFM1-R81C differing by R81 (blue) and C81 (red), respectively. Each sample was analyzed with two different LC-MS/MS methods (bars at 1, and 2, see Methods section for details). Additional analysis with 2nd LC-MS/MS method and 2x larger amount of injected sample was needed to identify C-terminal peptide of UFM1-RC (bar at 3). The diagram shows the number of MS/MS scans identified as the respective C-terminal peptide (peptide-spectrum matches, PSMs) in the individual samples. The PSMs for the different Cys-variants of the mutated peptide (see above) were summed up.

(D) Relative comparison of UFM1 abundance estimated via label-free protein quantification according to the TopN approach. The quantification was based on the four most abundant (Top4) peptides, unmodified and shared by both UFM1 variants ([K].ITLTSDPRLPYK.[V], [K].VLSVPESTPFTAVLK.[F], [K].FAAEEFK.[V], and [K].VPAATSAIITNDGIGINPAQTAGNVFLK.[H]; highlighted in grey in (B)). Each sample was analyzed with two different LC-MS/MS used also in (C). Note that UFM1-R81C is expressed at a level comparable to that of UFM1-WT.

(E, F) Fragment ion mass spectra of the C-terminal peptide of UFM1-WT (E, top panel) and UFM1-RC (N-ethylmaleimide-modified form, F, bottom panel). The respective presence of R81 or C81 was unambiguously confirmed by N- and C-terminal ion series. Only b- (red) and y-ions (blue) are labeled for clarity.

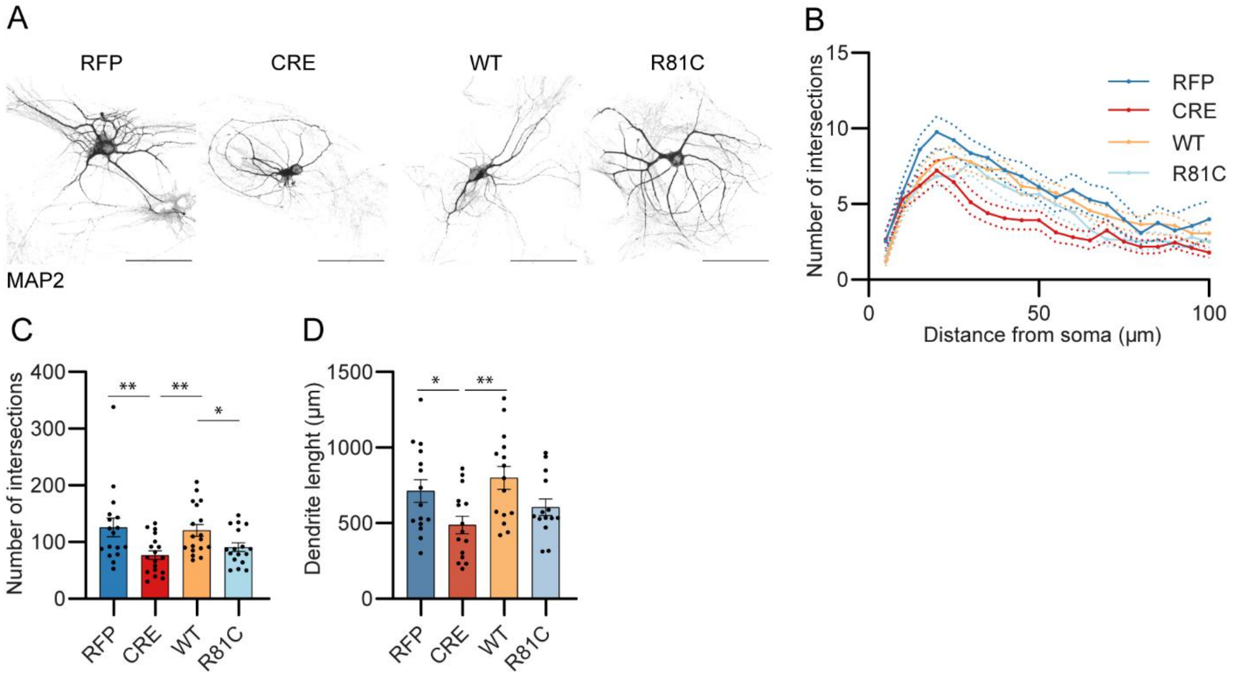

Appendix Figure S12: Neuronal expression of UFM1-R81C does not fully rescue neuronal complexity *in vitro*.

(A) Representative images of primary UFM1-cKO hippocampal neurons, infected at DIV 1 with viruses expressing RFP, CRE, CRE combined with UFM1-WT (WT) or UFM1-R81C (R81C), fixed and immunolabelled for MAP2 at DIV 5 (black). Scale bar, 50 µm.

(B) Sholl analysis line graph depicting the average number of dendrite intersections with concentric circles every 5 µm from the soma, using neuronal traces as shown in (A).

(C) Bar graph showing the total number of dendrite intersections, obtained from the Sholl analysis in (B).

(D) Bar graph showing the dendrite lengths, obtained from traces in (A).

For B-D, data were obtained from N = 3 independent experiments, n = 17 RFP, 18 CRE, 18 UFM1-WT (WT), and 17 UFM1-R81C (R81C) neurons. **, p<0.01 using a D'Agostino-Pearson normality test and Holm-Šídák's multiple comparisons test (C). *, p<0.05 using a D'Agostino-Pearson normality test and Mann Whitney test (D). Dotted lines and bar graphs show mean ± SEM.

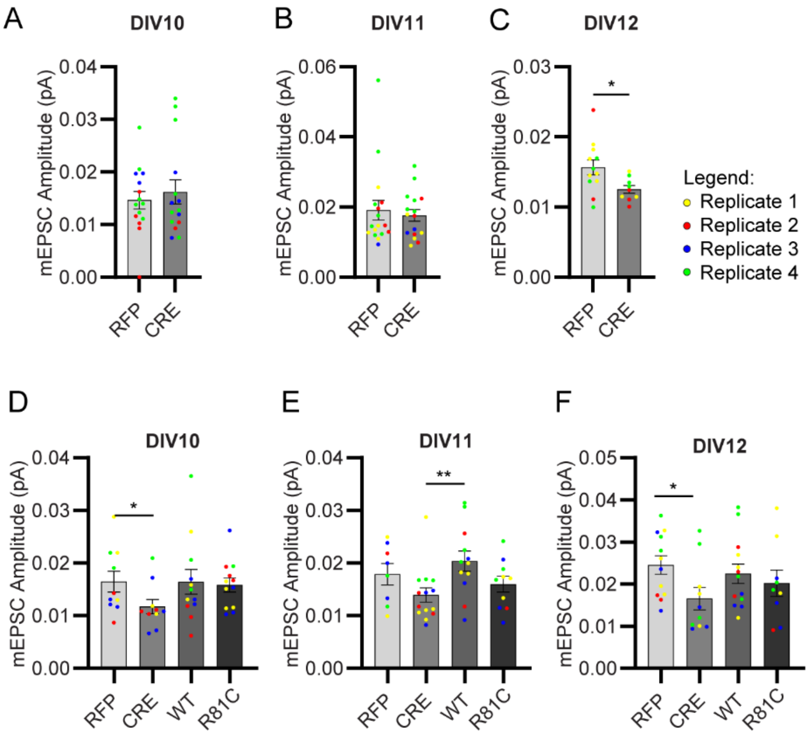

Appendix Figure S13: Variation in mEPSC amplitudes during autaptic culture maturation

(A-C) Bar graph depicting the mEPSC amplitudes of the different replicates used for Figure 3 (identified by the colours yellow, red, blue and green) of neurons after DIV 10 (A), DIV 11 (B) and DIV 12 (C). N=4 experiments, n=40 RFP cells and n=40 CRE cells. *, p<0.05 using a D'Agostino-Pearson normality test and Mann-Whitney test. Bar graphs show mean ± SEM.

(D-E) Bar graph depicting the mEPSC amplitudes of the different replicates used for Figure 5 (identified by the colours yellow, red, blue and green) of neurons after DIV 10 (D), DIV 11 (E) and DIV 12 (F). N=3 independent experiments where n= 23 RFP, 26 CRE, 26 UFM1-WT (WT) and 26 UFM1-R81C (R81C) cells. **, p<0.01, *, p<0.05 using a D'Agostino-Pearson normality test and Kruskal-Wallis test. Bar graphs show mean ± SEM.

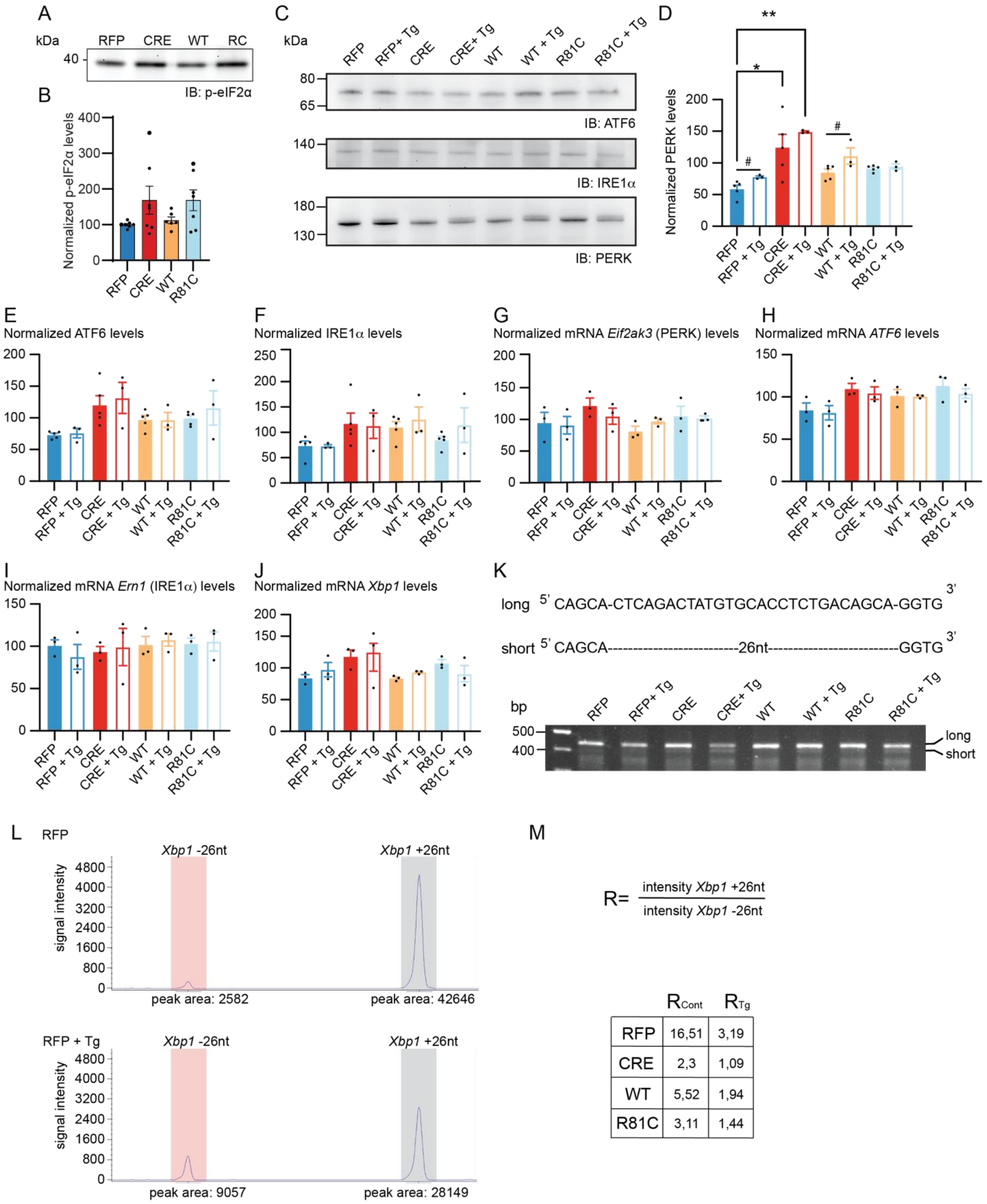

	R_{Cont}	R_{Tg}
RFP	16,51	3,19
CRE	2,3	1,09
WT	5,52	1,94
R81C	3,11	1,44

Appendix Figure S14: Characterization of the UPR response.

(A) Anti-p-eIF2α IB analysis of lysates from DIV 13 UFM1-cKO primary hippocampal neurons infected at DIV 1 with viruses expressing RFP, CRE, CRE combined with UFM1-WT (WT) or UFM1- R81C (R81C). Molecular weights are indicated on the left (kDa).

(B) Bar graph depicting the quantification of p-eIF2α IB signal as shown in A. N=4 experiments, ns $p>0.1$ using a D'Agostino-Pearson normality test and ordinary one-way ANOVA. Bar graphs show mean ± SEM.

(C) Representative anti-cleaved ATF6 (top), anti-IRE1α (middle) and anti-PERK (bottom) Western blot analysis of DIV 10 lysates of neurons infected at DIV 1 with viruses expressing either RFP, CRE, CRE combined with UFM1-WT or UFM1-R81C. Shortly before lysis, neurons were treated for 15min with 3μM of thapsigargin (+Tg) or DMSO as control (Cont).

(D-F) Bar graph showing the quantification of the immunosignal observed in C. N=3 experiments, *, $p=0.0267$; **, $p=0.0026$ using an ordinary one way ANOVA followed by Kruskal-Wallis test. # $p<0.05$ using a Mann-Whtiney test. Bar graph shows mean +/- SEM.

(G-J) Bar graph showing the normalized mRNA levels for *Eif2ak3* (PERK, G), *ATF6* (H), *Ern1* (IRE1α, I) and *Xbp1* (J) as measured via qPCR.

(K) Nucleotides (nt) sequences of mouse cDNA corresponding to uncleaved (long, top lane) and cleaved (short, bottom panel) *Xbp1* mRNA. Representative agarose gel image of *Xbp1* qPCR fragment analysis. Long, uncleaved *Xbp1* variant; short, cleaved *Xbp1* variant; bp, base pair marker.

(L) Typical electropherogram analysis of *Xbp1* cleavage in control (Cont) and thapsigargin (Tg) treated neurons. The area of each peak is indicated below.

(M) Ratiometric measurement of the area below peak between the long and short *Xbp1* fragment as shown in L.

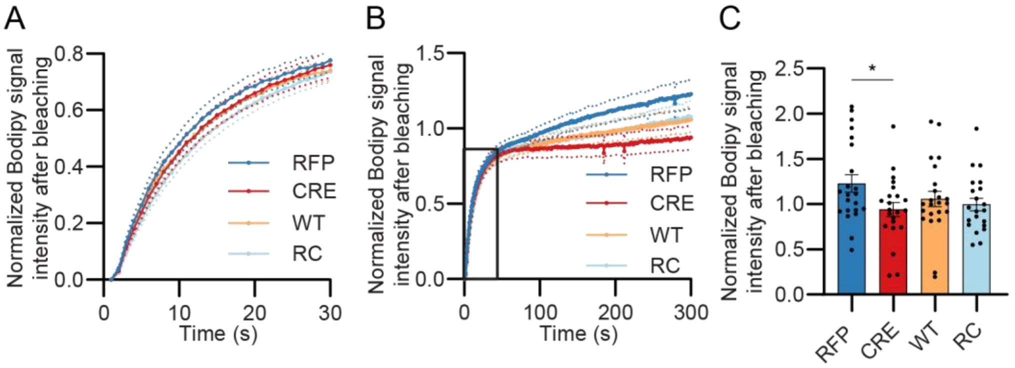

Appendix Figure S15: Neuronal expression of UFM1-R81C does not change the ER membrane fluidity

(A, B) Line graphs showing the quantification of Bodipy signal intensity recovery over time after bleaching (0 s) and up until 30 s (A) or until 300 s (B). UFM1-cKO hippocampal neurons were infected at DIV 1 with RFP, CRE, CRE combined with UFM1-WT (WT) or UFM1-R81C (R81C) expressing viruses, incubated with Bodipy (3μM, 15min) and imaged. The black square indicates the region enlarged in (A). Dotted lines show mean ± SEM.

(C) Bar graph showing the normalized Bodipy intensity 300s after bleaching. Bar graph shows means ± SEM. Data were obtained from N=3 experiments where n= 22 RFP, 22 CRE, 23 UFM1-WT (WT) and 21 UFM1-R81C (R81C) cells. *, $p<0.05$ using a D'Agostino-Pearson normality test and Mann Whitney test.

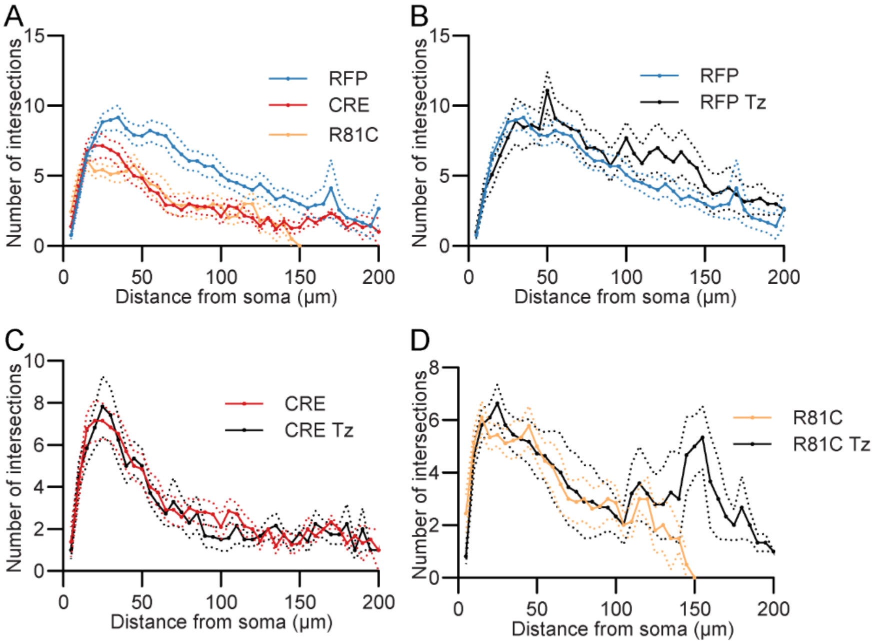

Appendix Figure S16: Trazodone treatment does not alter neuronal morphology

(A-D) Sholl analysis line graph of primary hippocampal UFM1-cKO neurons infected at DIV 1 with viruses expressing RFP, CRE or CRE combined with UFM1-R81C (R81C), cultured for 12 days, treated with Trazodone (Tz, 20 µM) or DMSO as a control every 3 days. Dotted lines show mean ± SEM.

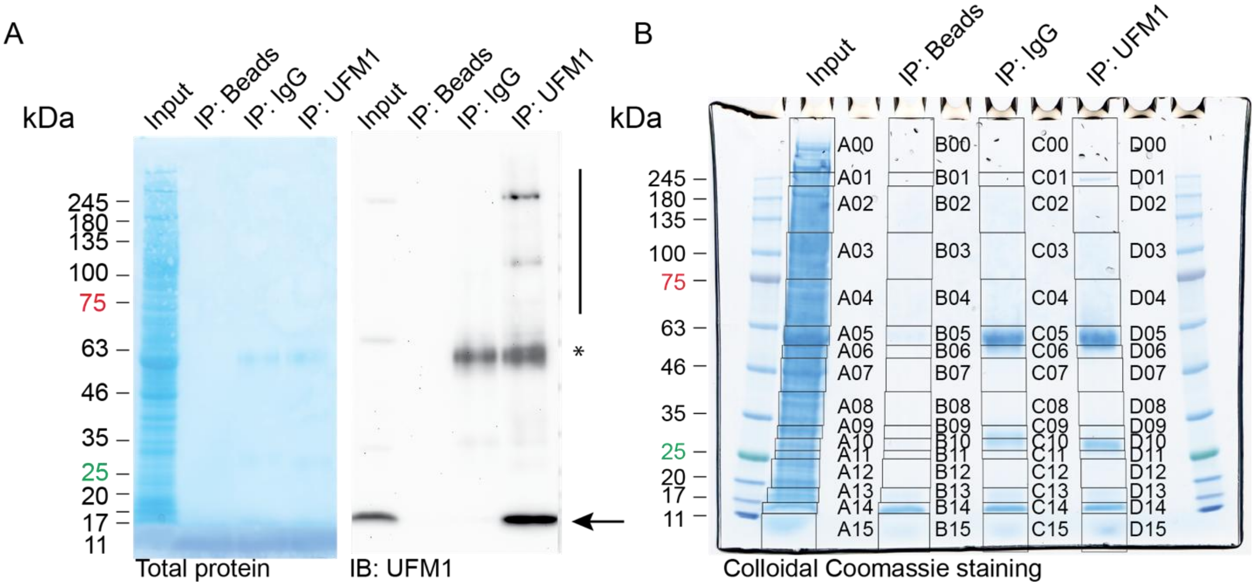

C

UFM1 (P61961)

Gel band number	Input (A)	IP Beads (B)	IP IgG (C)	IP UFM1 (D)
00	0	0	0	0
01	0	0	0	2
02	1	0	0	8
03	0	0	0	5
04	0	0	0	9
05	1	0	0	1
06	0	0	0	1
07	0	0	0	6
08	0	0	0	23
09	1	0	0	7
10	1	0	0	3
11	0	0	0	0
12	0	0	0	0
13	1	0	0	9
14	8	0	0	37
15	0	0	0	0

UBA5 (Q8VE47)

Gel band number	Input (A)	IP Beads (B)	IP IgG (C)	IP UFM1 (D)
00	0	0	0	0
01	0	0	0	0
02	0	0	0	3
03	0	0	0	7
04	0	0	0	39
05	1	0	0	29
06	12	0	0	96
07	10	0	0	31
08	0	0	0	2
09	0	0	0	0
10	0	0	0	0
11	0	0	0	0
12	0	0	0	0
13	0	0	0	0
14	0	0	0	0
15	0	0	0	0

UFC1 (Q9CR09)

Gel band number	Input (A)	IP Beads (B)	IP IgG (C)	IP UFM1 (D)
00	0	0	0	0
01	0	0	0	0
02	0	0	0	0
03	0	0	0	0
04	0	0	0	0
05	0	0	0	0
06	0	0	0	1
07	0	0	0	3
08	0	0	0	12
09	0	0	0	7
10	0	0	0	3
11	1	0	0	15
12	4	0	0	27
13	0	0	0	0
14	0	0	0	0
15	0	0	0	0

DOCK4 (P59764)

Gel band number	Input (A)	IP Beads (B)	IP IgG (C)	IP UFM1 (D)
00	3	0	0	117
01	104	0	0	860
02	0	0	0	140
03	0	0	0	118
04	0	0	0	31
05	0	0	0	1
06	0	0	0	5
07	0	0	0	19
08	0	0	0	0
09	0	0	0	0
10	0	0	0	0
11	0	0	0	0
12	0	0	0	0
13	0	0	0	0
14	0	0	0	0
15	0	0	0	0

Appendix Figure S17: Identification of mouse brain UFM1 candidate targets

(A, B) Total protein stain (A, left), anti-UFM1 Western blot analysis (A, right) and Colloidal Coomassie stained gel (B) of input and eluate fractions from anti-UFM1 affinity purification from mouse brain. The black arrow indicates free, unconjugated UFM1, the asterisk indicates IgG heavy chains and the black line indicates specific anti-UFM1 signals. Purifications performed with beads only and non-specific IgG affinity were used as negative controls. In B, bands excised for tryptic digest are marked A00 to D15.

(C) Heat map table indicating number of peptide-spectrum-matches (PSM) identified by LC-MS/MS as a proxy for the abundance of selected proteins in corresponding gel pieces (UFM1, theoretical molecular weight 9.1 kDa; UBA5, 44.8kDa; UFC1, 19.5 kDa; DOCK4, 226kDa).
