# Supplementary figures and images for: Encephalopathy-linked UFM1 variants impede neuronal protein translation, development, and function
Source: EMBO Mol Med. 2026 Feb 23;18(4):1265–91. doi: 10.1038/s44321-026-00389-6 (PMC13083916; doi:10.1038/s44321-026-00389-6)

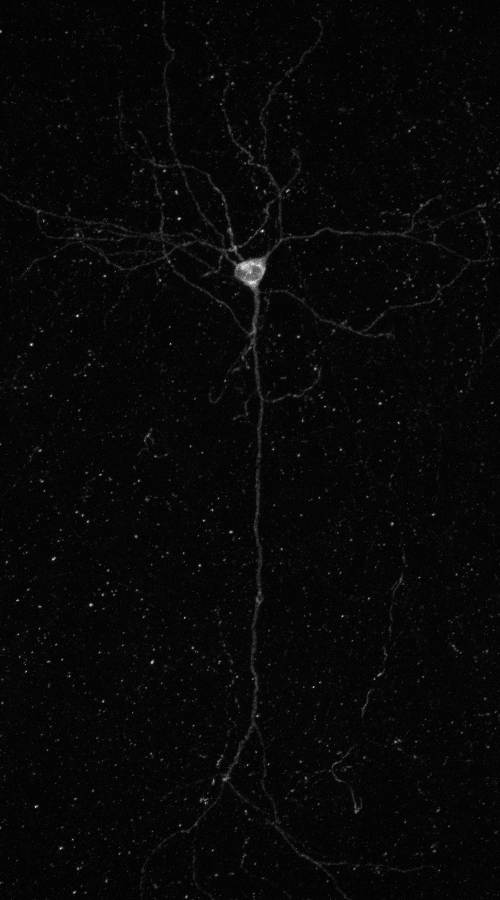

Supplement: Supplementary file 5 — Source data Fig. 1 [file 44321_2026_389_MOESM5_ESM.zip › Figure_1/1A/WT.tif]

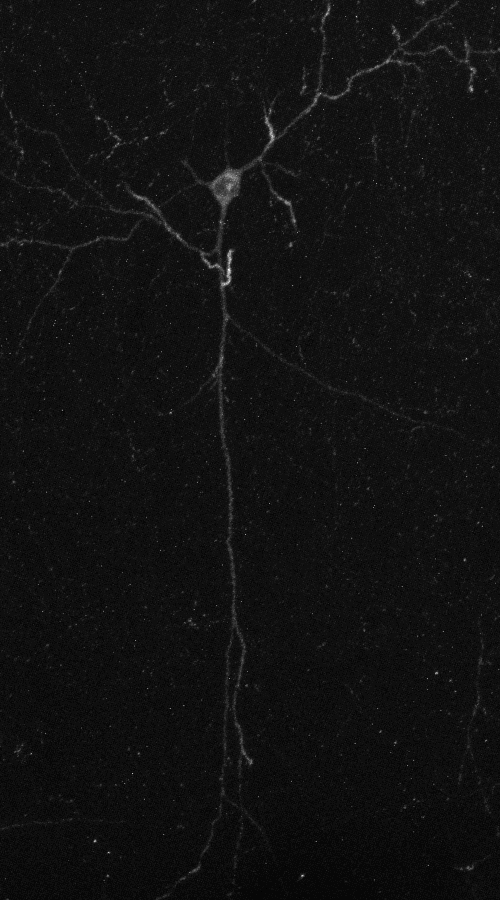

Supplement: Supplementary file 5 — Source data Fig. 1 [file 44321_2026_389_MOESM5_ESM.zip › Figure_1/1A/KO.tif]

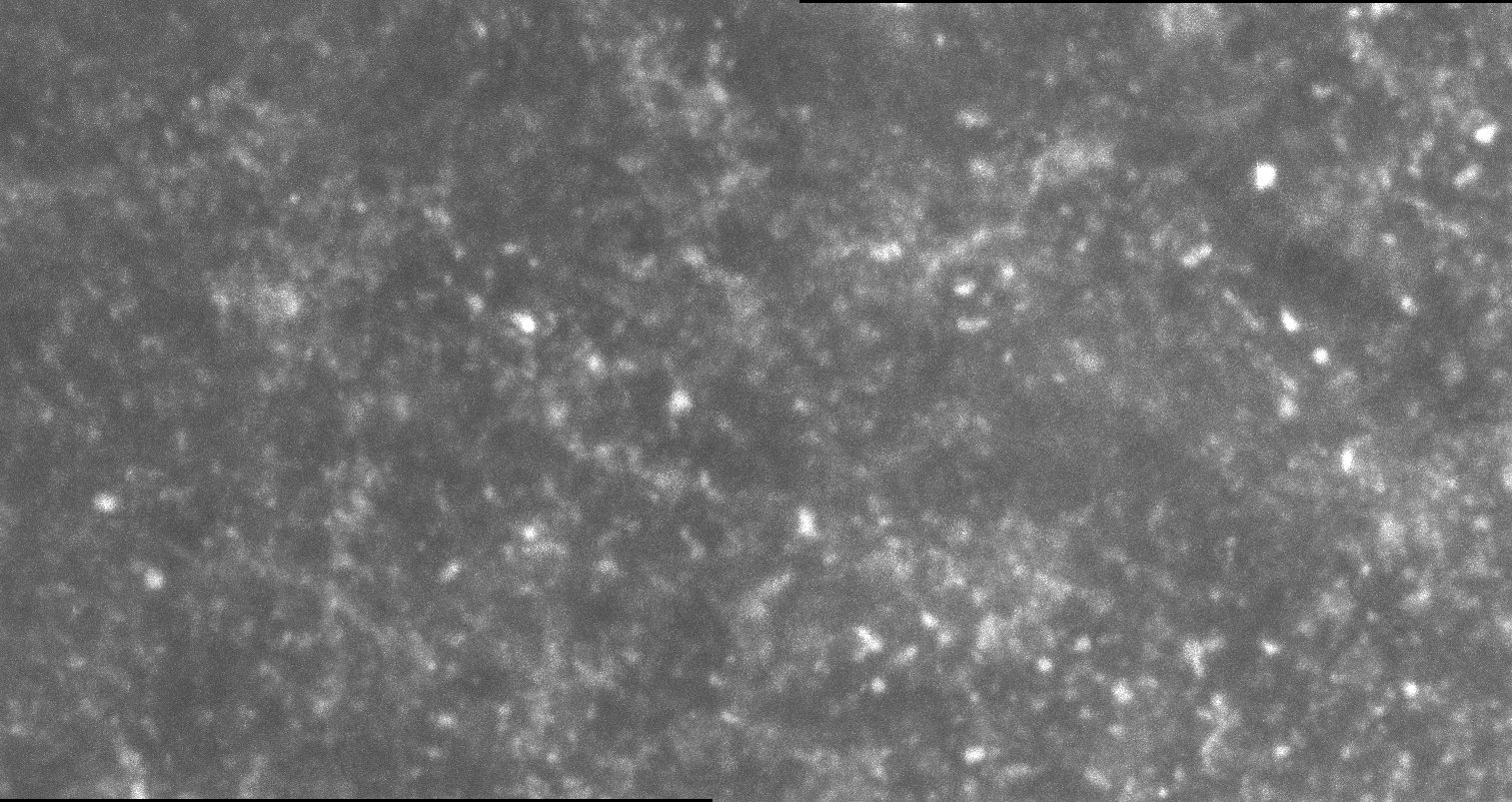

Supplement: Supplementary file 5 — Source data Fig. 1 [file 44321_2026_389_MOESM5_ESM.zip › Figure_1/1E/WT.tif]

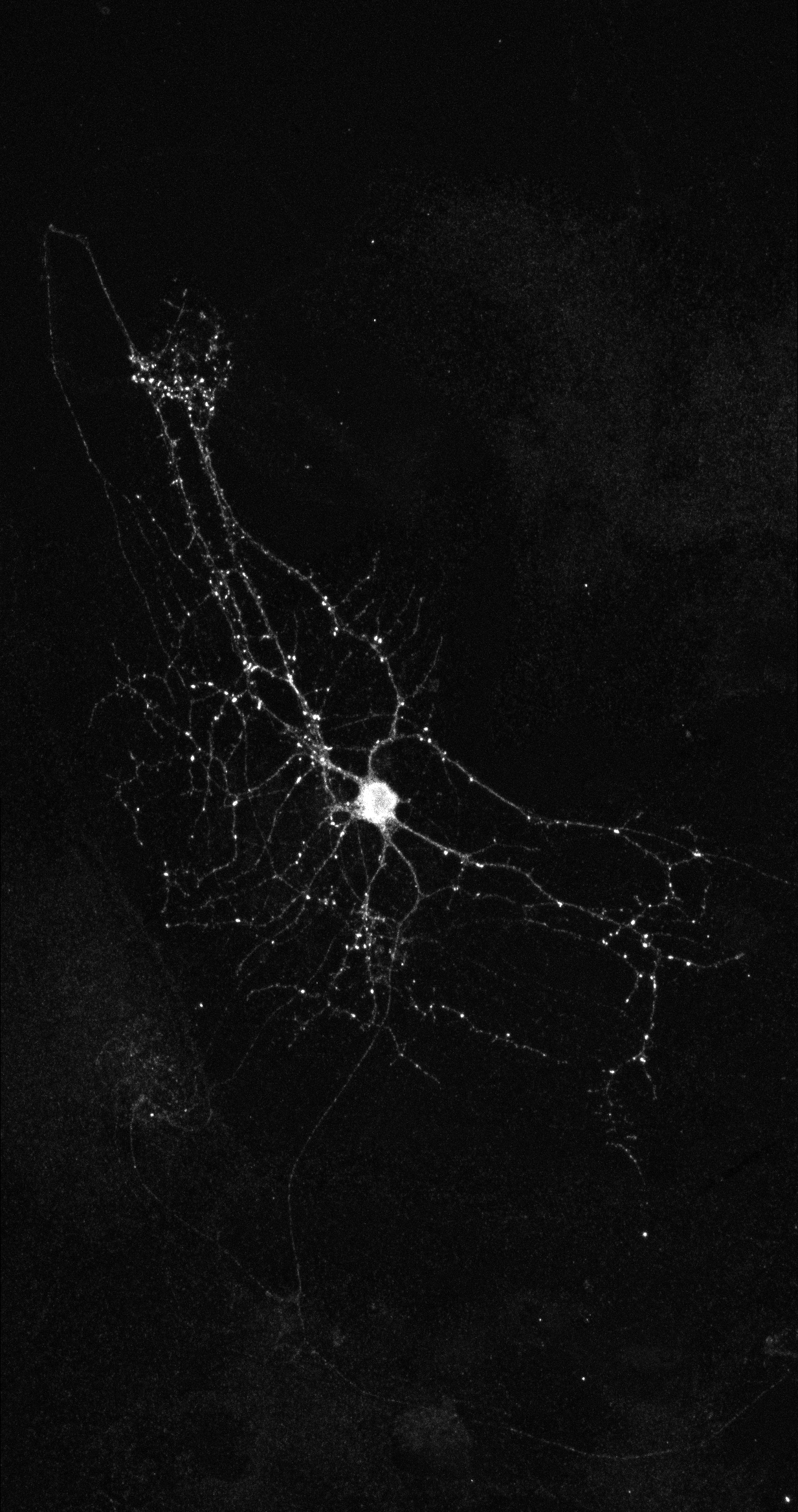

Supplement: Supplementary file 6 — Source data Fig. 2 [file 44321_2026_389_MOESM6_ESM.zip › Figure_2/2D/RFP.tif]

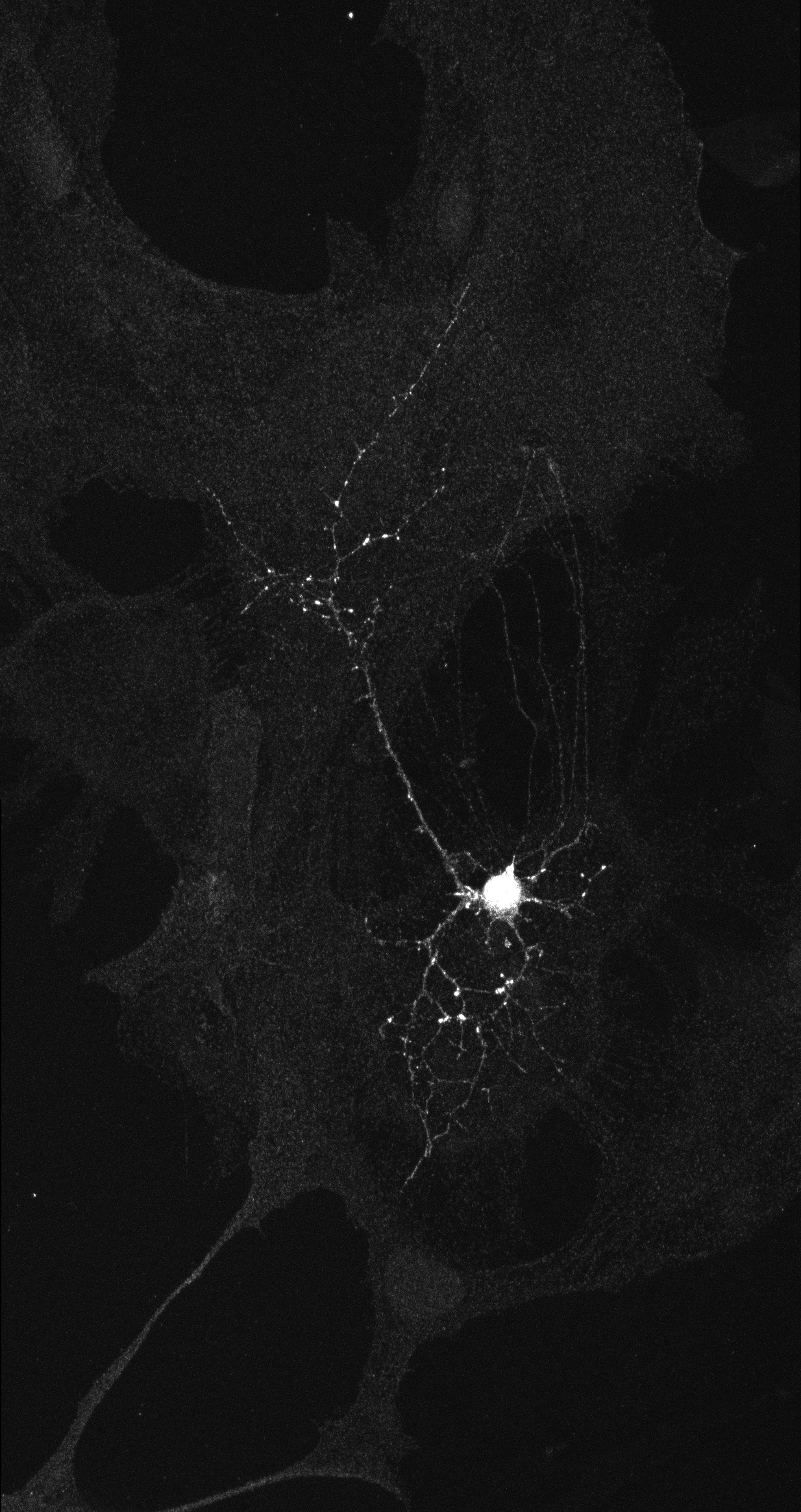

Supplement: Supplementary file 6 — Source data Fig. 2 [file 44321_2026_389_MOESM6_ESM.zip › Figure_2/2D/CRE.tif]

Figure 2 B

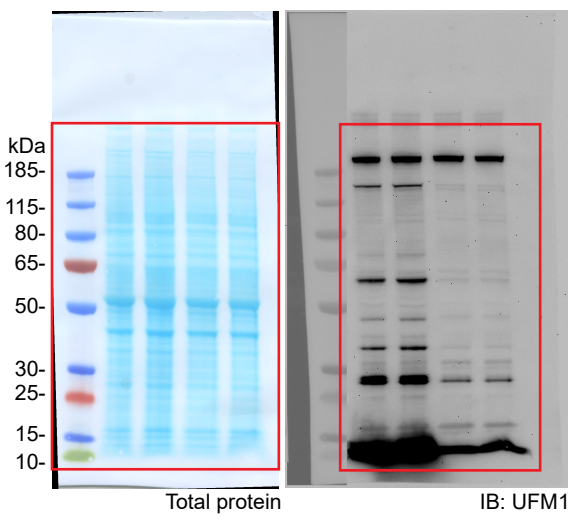

Supplement: Supplementary file 6 — Source data Fig. 2 [file 44321_2026_389_MOESM6_ESM.zip › Figure_2/2B/blot_2B.pdf]

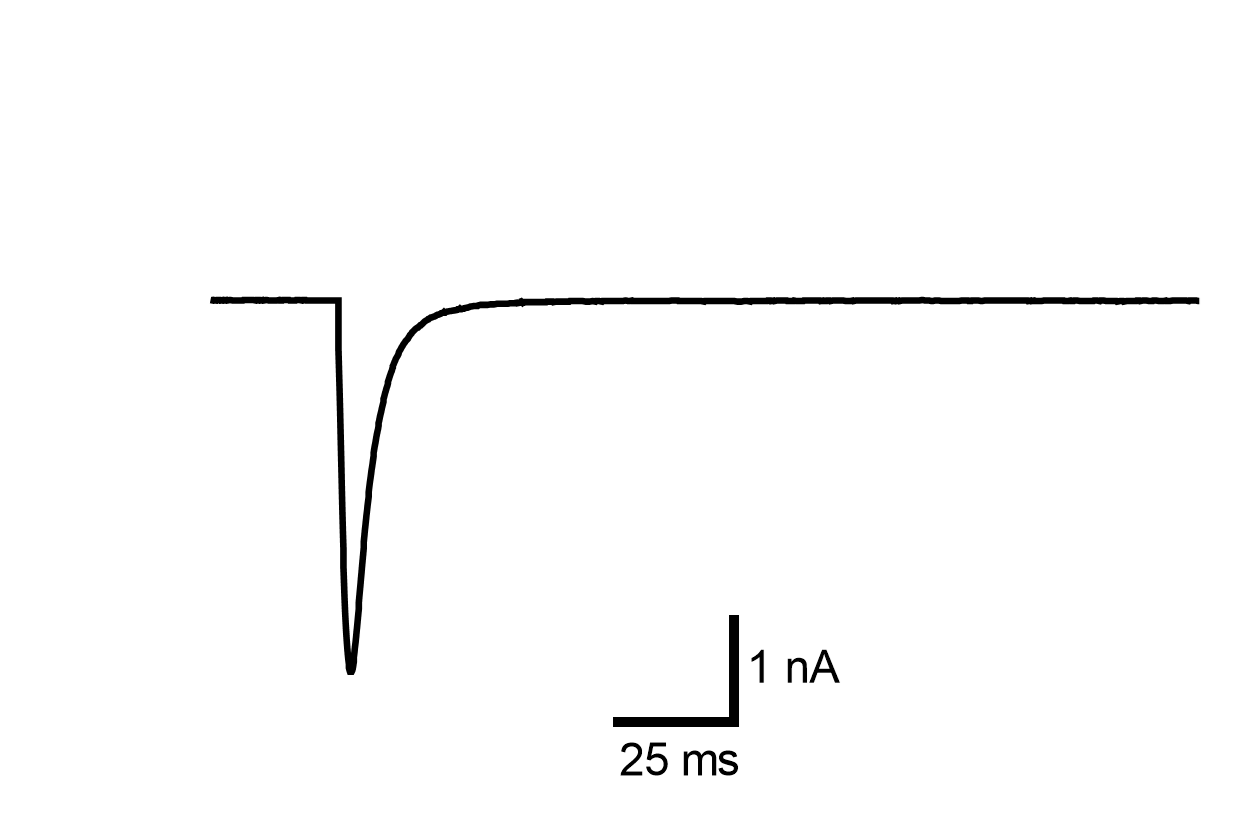

Supplement: Supplementary file 7 — Source data Fig. 3 [file 44321_2026_389_MOESM7_ESM.zip › Figure_3/3A/RFP_EPSC.tif]

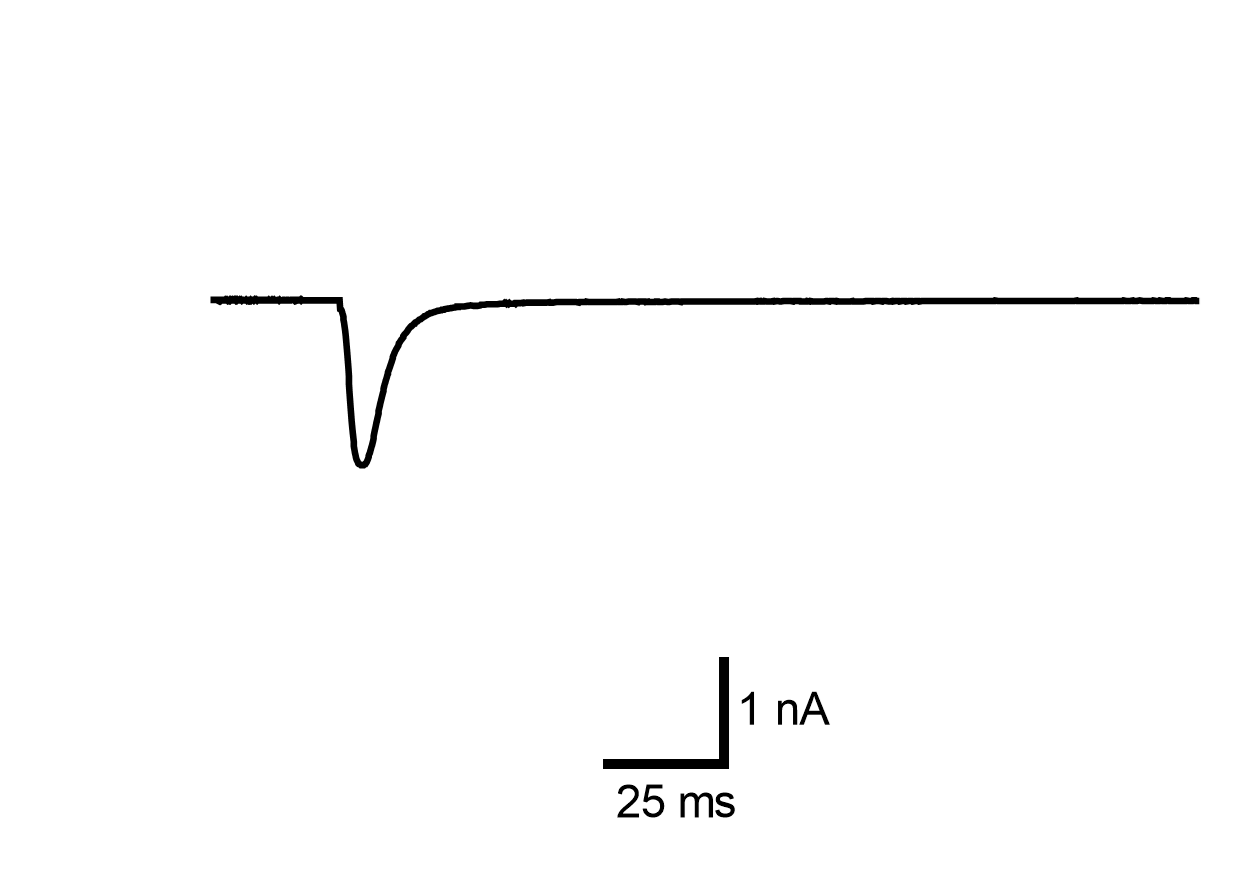

Supplement: Supplementary file 7 — Source data Fig. 3 [file 44321_2026_389_MOESM7_ESM.zip › Figure_3/3A/CRE_EPSC.tif]

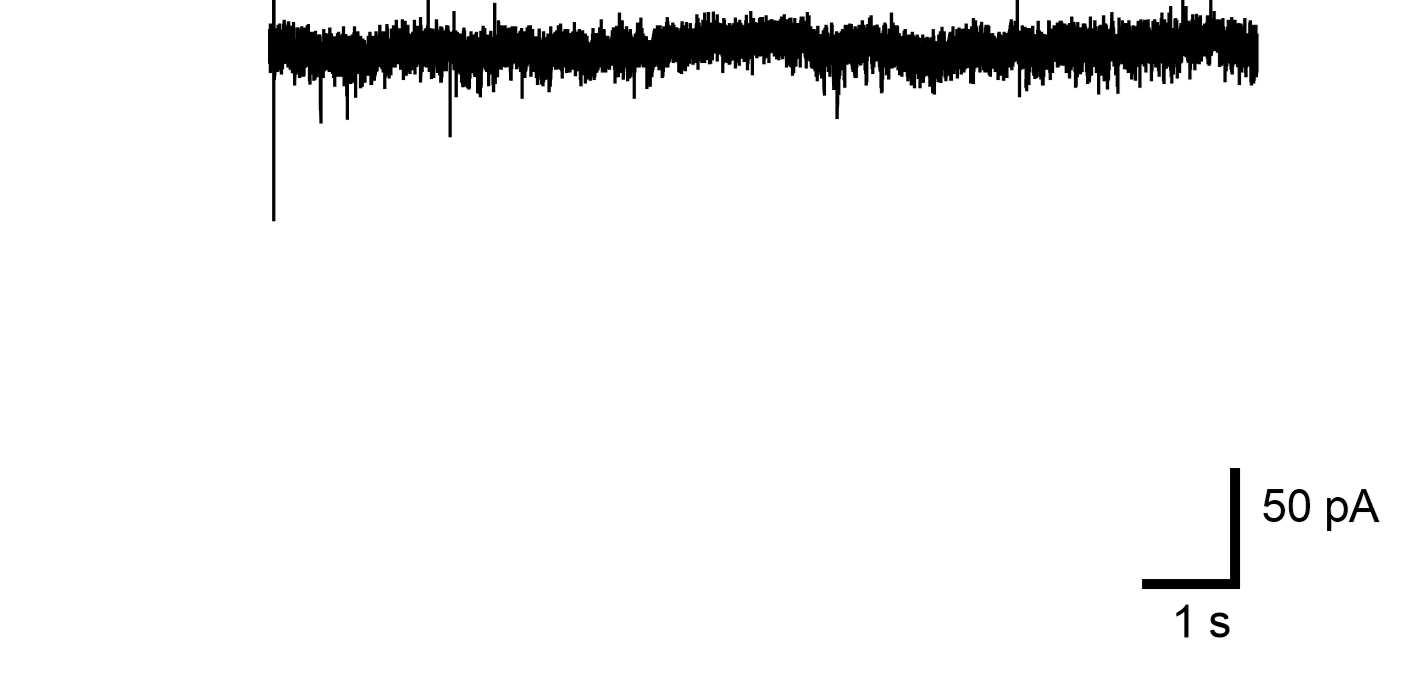

Supplement: Supplementary file 7 — Source data Fig. 3 [file 44321_2026_389_MOESM7_ESM.zip › Figure_3/3F/mEPSC_CRE.tif]

Figure 4 C

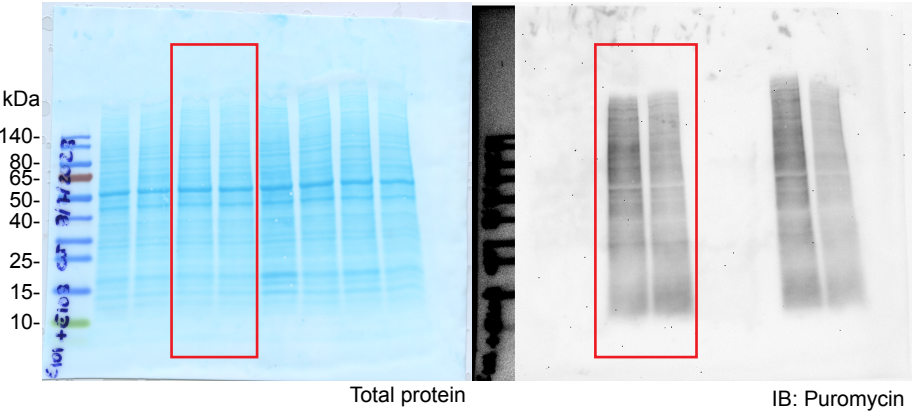

Supplement: Supplementary file 8 — Source data Fig. 4 [file 44321_2026_389_MOESM8_ESM.zip › Figure_4/4C/Original_blots 4C.pdf]

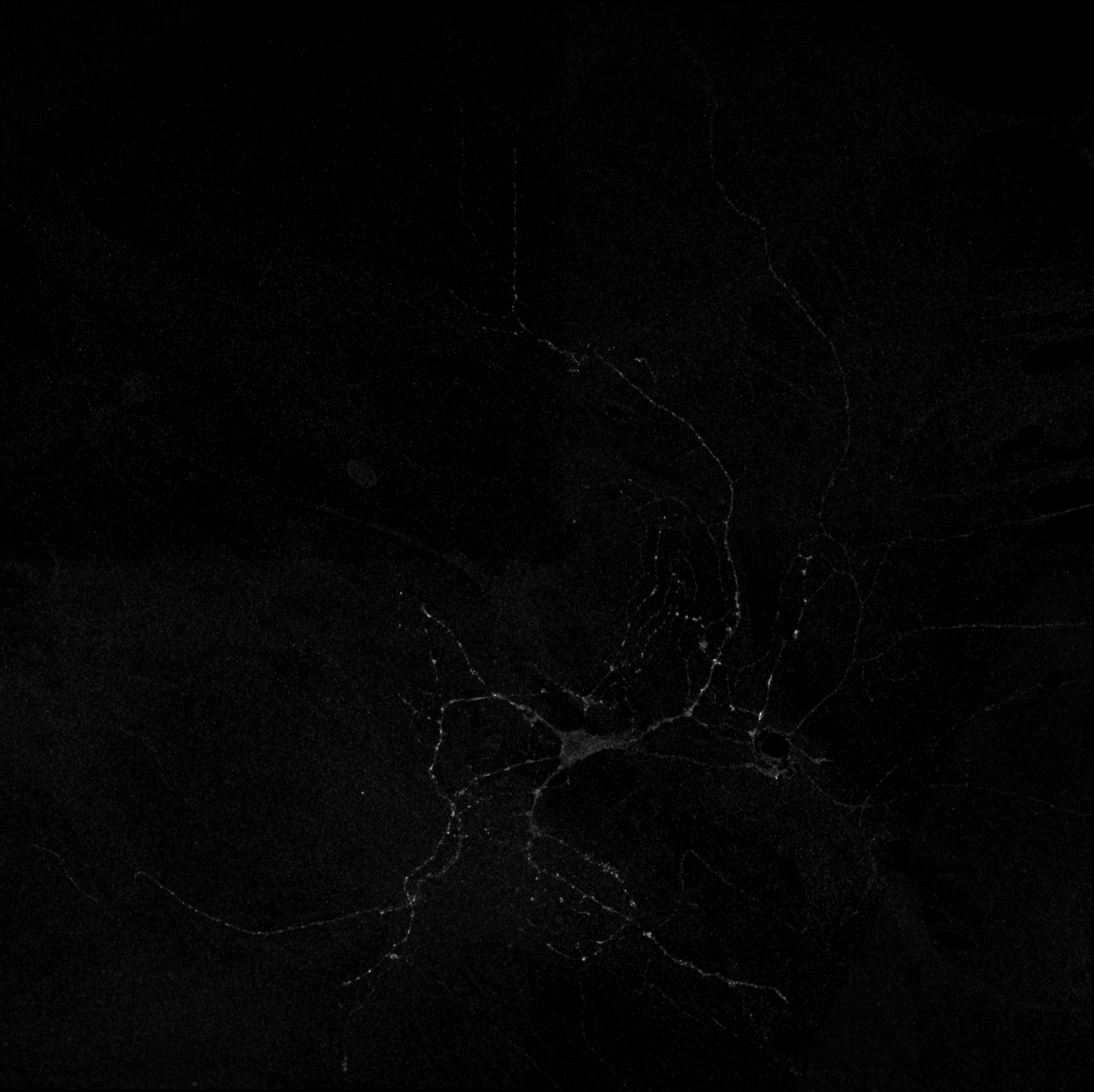

Supplement: Supplementary file 9 — Source data Fig. 5 [file 44321_2026_389_MOESM9_ESM.zip › Figure_5/5A/RFP.tif]

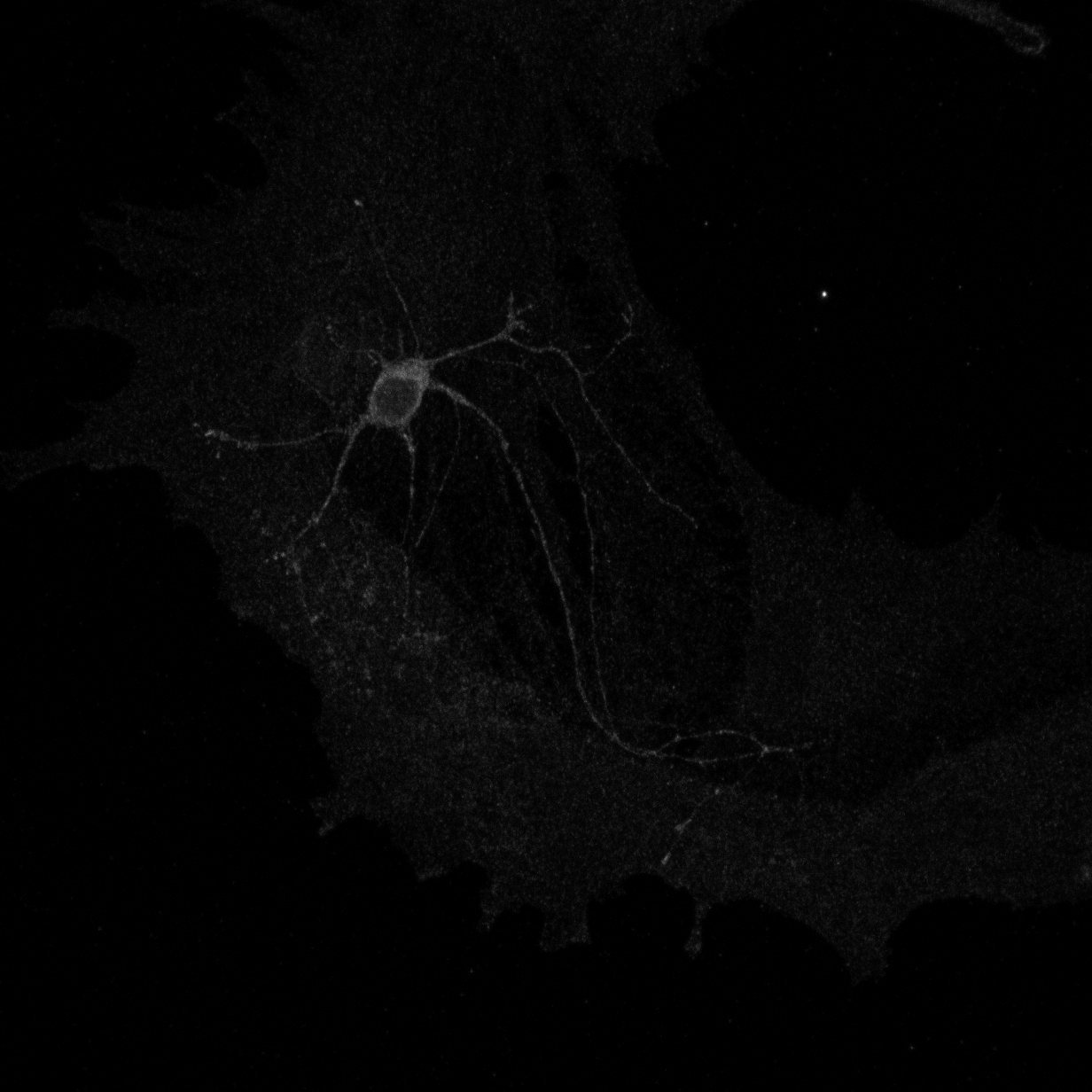

Supplement: Supplementary file 9 — Source data Fig. 5 [file 44321_2026_389_MOESM9_ESM.zip › Figure_5/5A/CRE.tif]

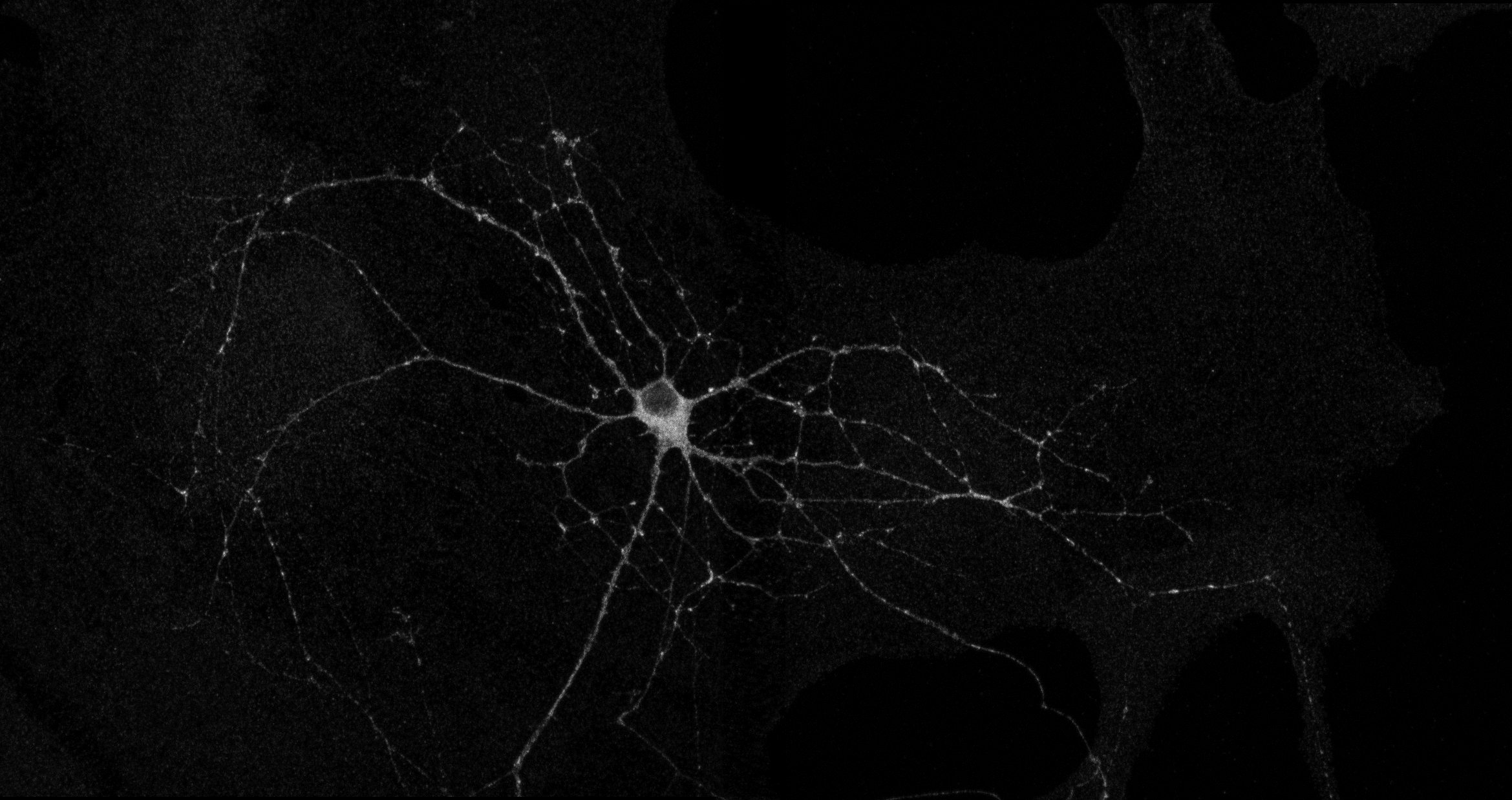

Supplement: Supplementary file 9 — Source data Fig. 5 [file 44321_2026_389_MOESM9_ESM.zip › Figure_5/5A/WT.tif]

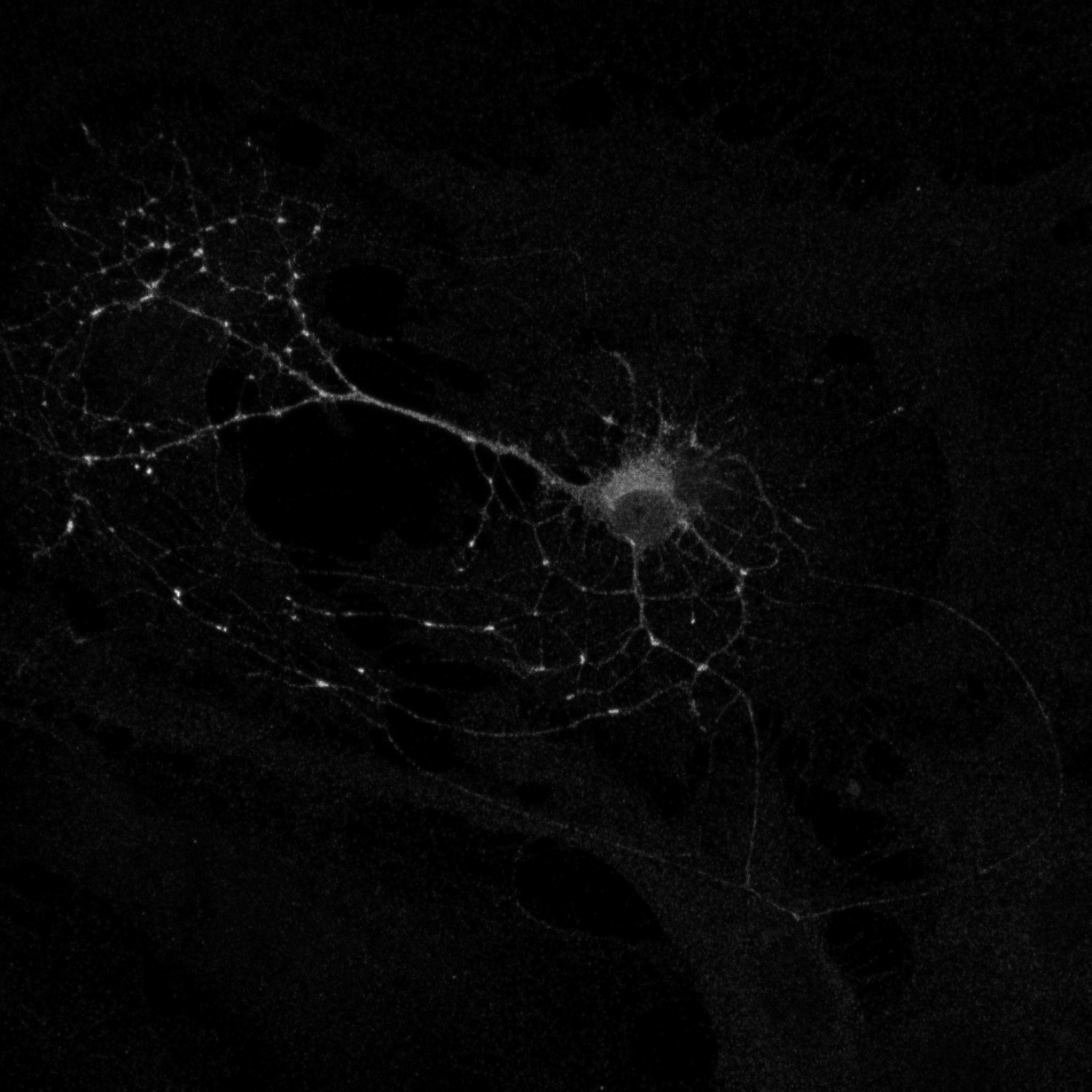

Supplement: Supplementary file 9 — Source data Fig. 5 [file 44321_2026_389_MOESM9_ESM.zip › Figure_5/5A/R81C.tif]

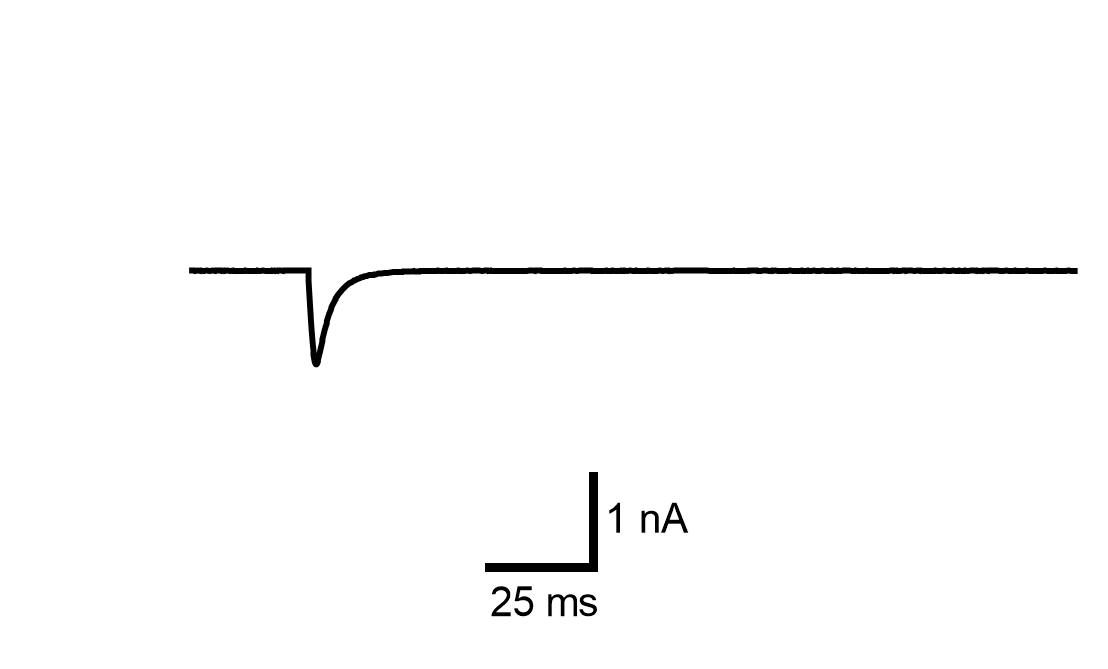

Supplement: Supplementary file 10 — Source data Fig. 6 [file 44321_2026_389_MOESM10_ESM.zip › Figure_6/6A/Rescue_EPSC_CRE-RC.tif]

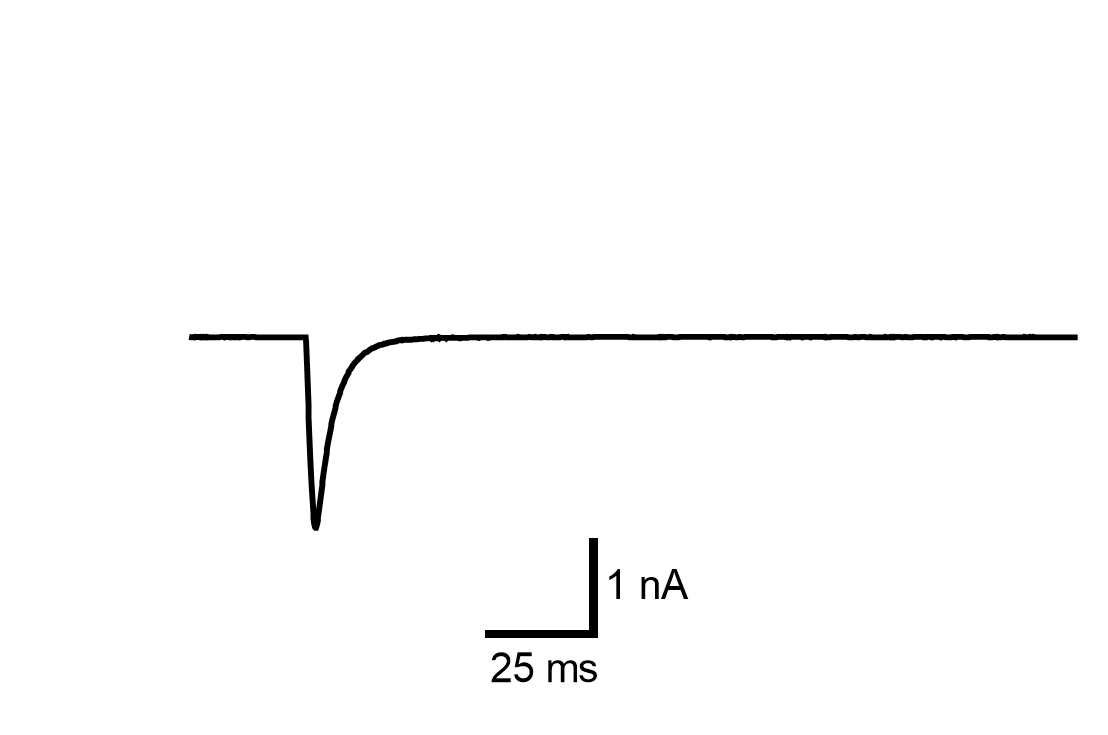

Supplement: Supplementary file 10 — Source data Fig. 6 [file 44321_2026_389_MOESM10_ESM.zip › Figure_6/6A/Rescue_EPSC_RFP.tif]

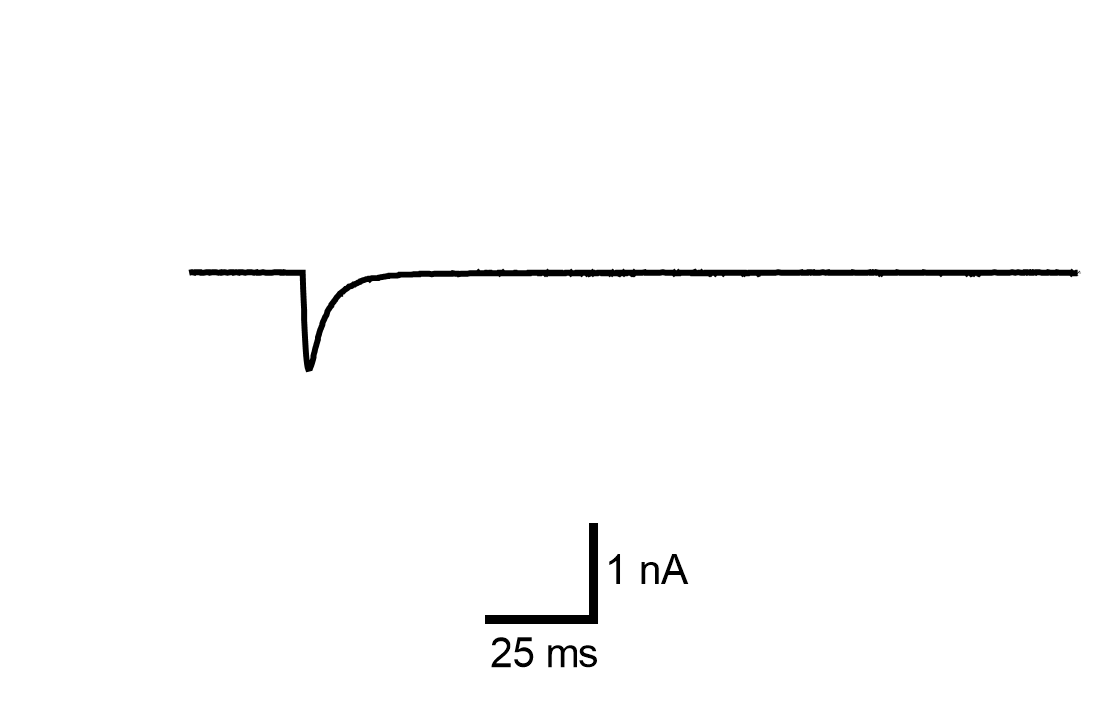

Supplement: Supplementary file 10 — Source data Fig. 6 [file 44321_2026_389_MOESM10_ESM.zip › Figure_6/6A/Rescue_EPSC_CRE-WT.tif]

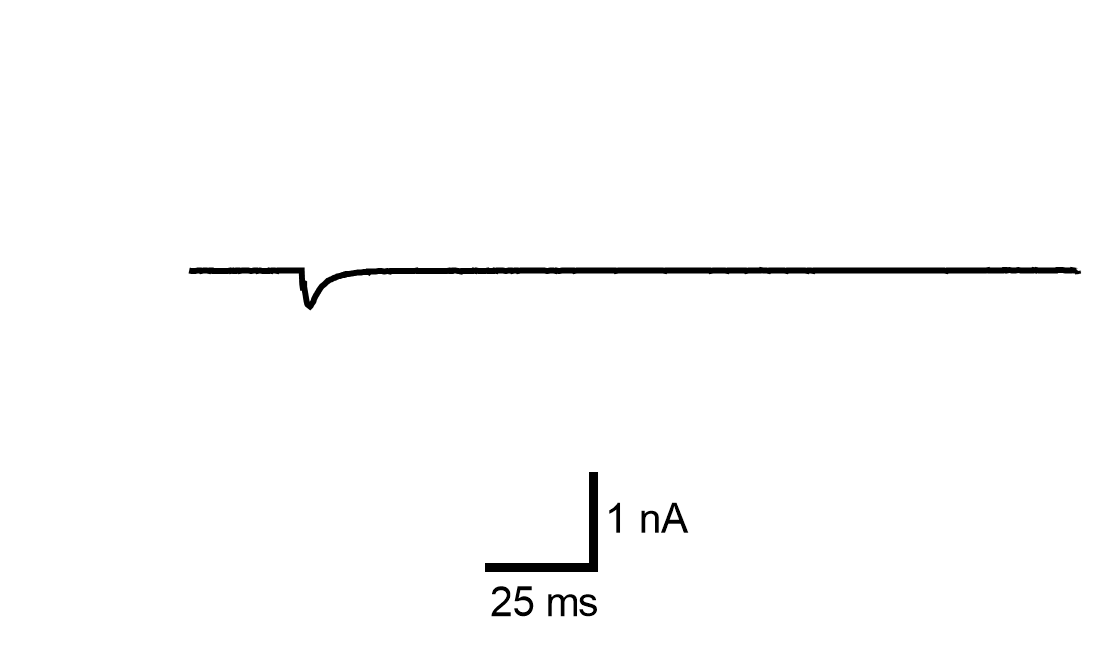

Supplement: Supplementary file 10 — Source data Fig. 6 [file 44321_2026_389_MOESM10_ESM.zip › Figure_6/6A/Rescue_EPSC_CRE.tif]

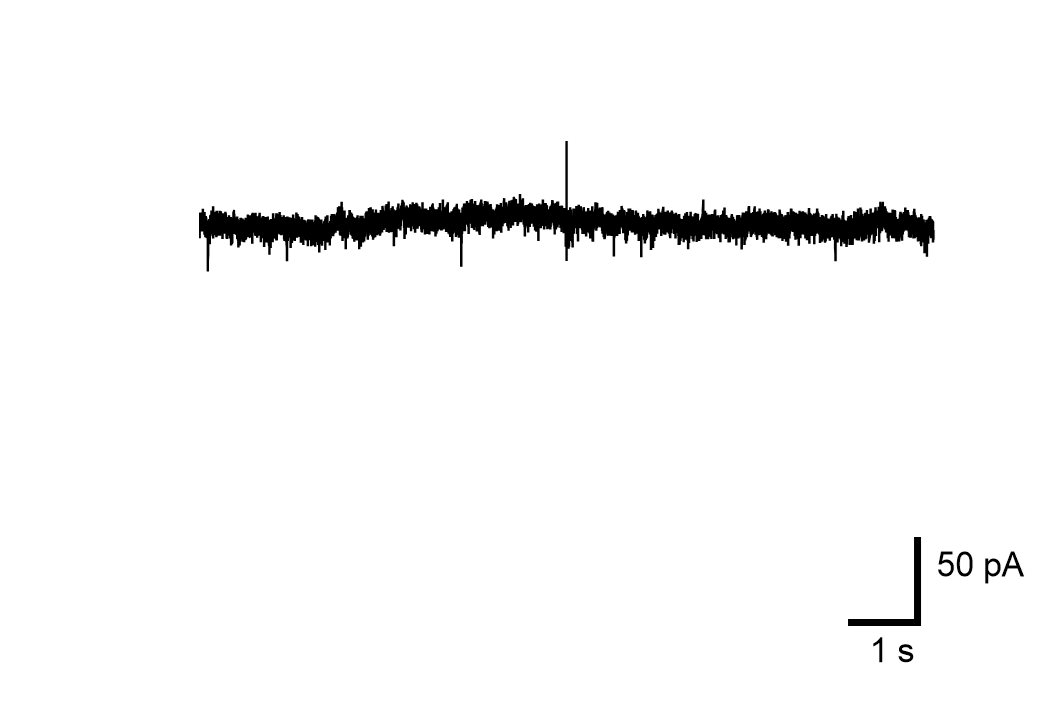

Supplement: Supplementary file 10 — Source data Fig. 6 [file 44321_2026_389_MOESM10_ESM.zip › Figure_6/6E/Rescue_CRE.tif]

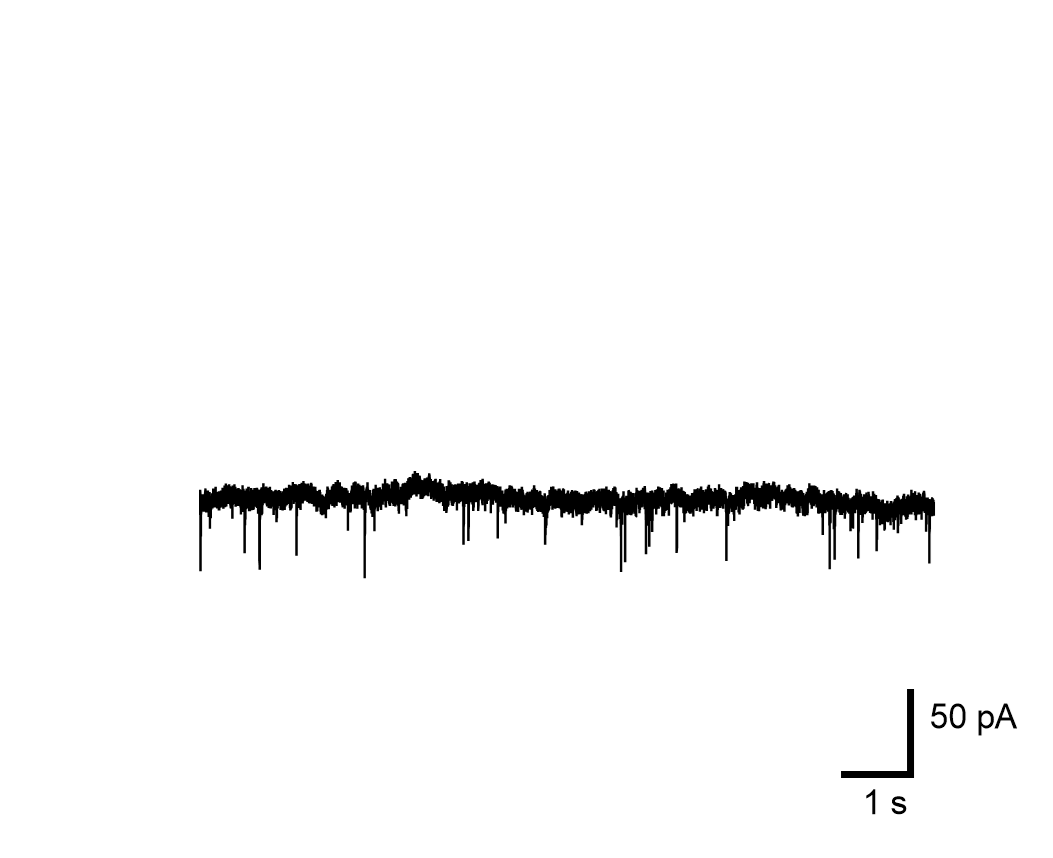

Supplement: Supplementary file 10 — Source data Fig. 6 [file 44321_2026_389_MOESM10_ESM.zip › Figure_6/6E/Rescue_CRE-WT.tif]

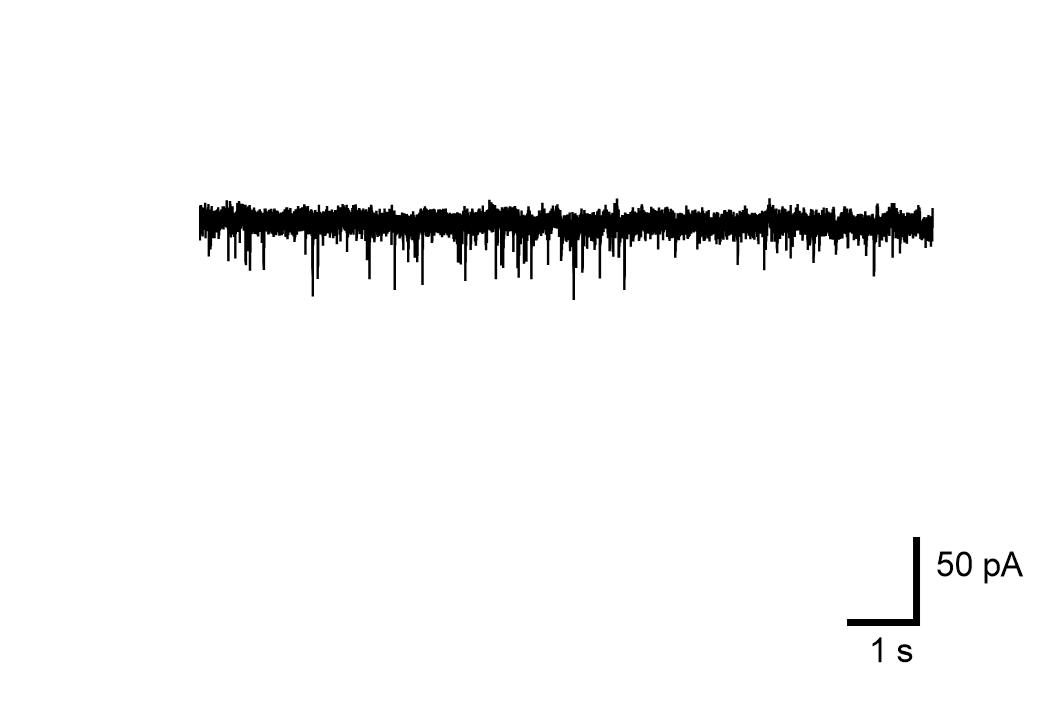

Supplement: Supplementary file 10 — Source data Fig. 6 [file 44321_2026_389_MOESM10_ESM.zip › Figure_6/6E/Rescue_RFP.tif]

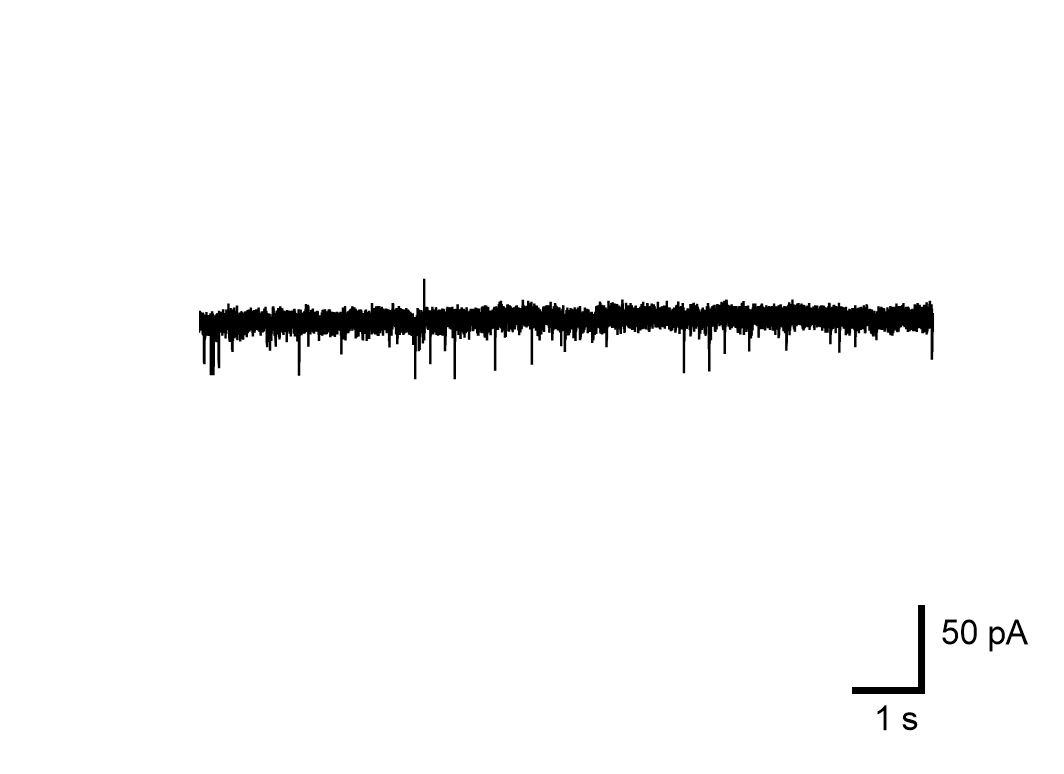

Supplement: Supplementary file 10 — Source data Fig. 6 [file 44321_2026_389_MOESM10_ESM.zip › Figure_6/6E/Rescue_CRE-RC.tif]

Figure 7 C  
ATF6

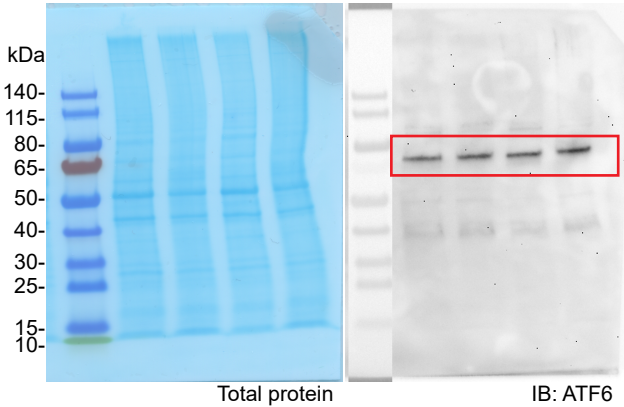

Figure 7 C  
IRE1 $\alpha$

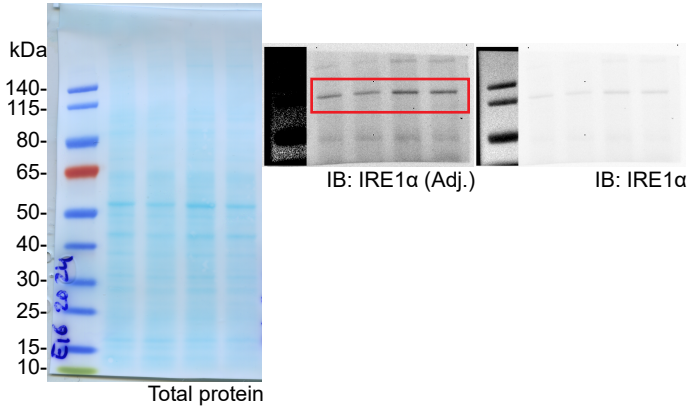

Figure 7 C  
PERK

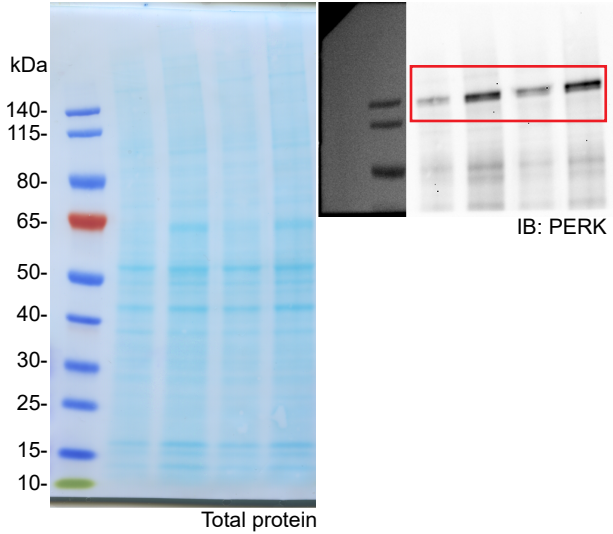

Supplement: Supplementary file 11 — Source data Fig. 7 [file 44321_2026_389_MOESM11_ESM.zip › Figure_7/7C/blots_7C.pdf]

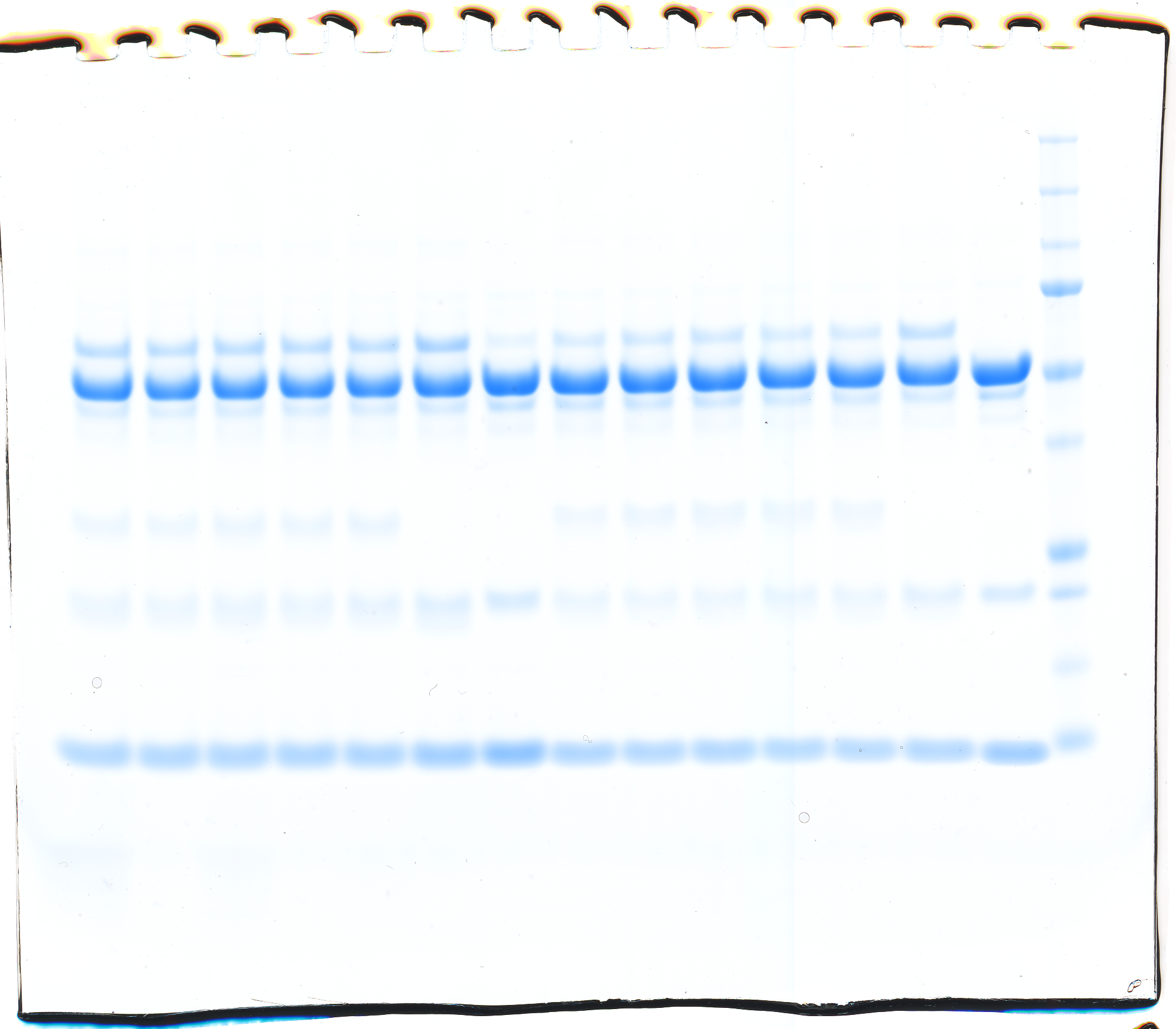

Supplement: Supplementary file 12 — Source data Fig. 8 [file 44321_2026_389_MOESM12_ESM.zip › Figure_8/8F/Figure8F.tif]

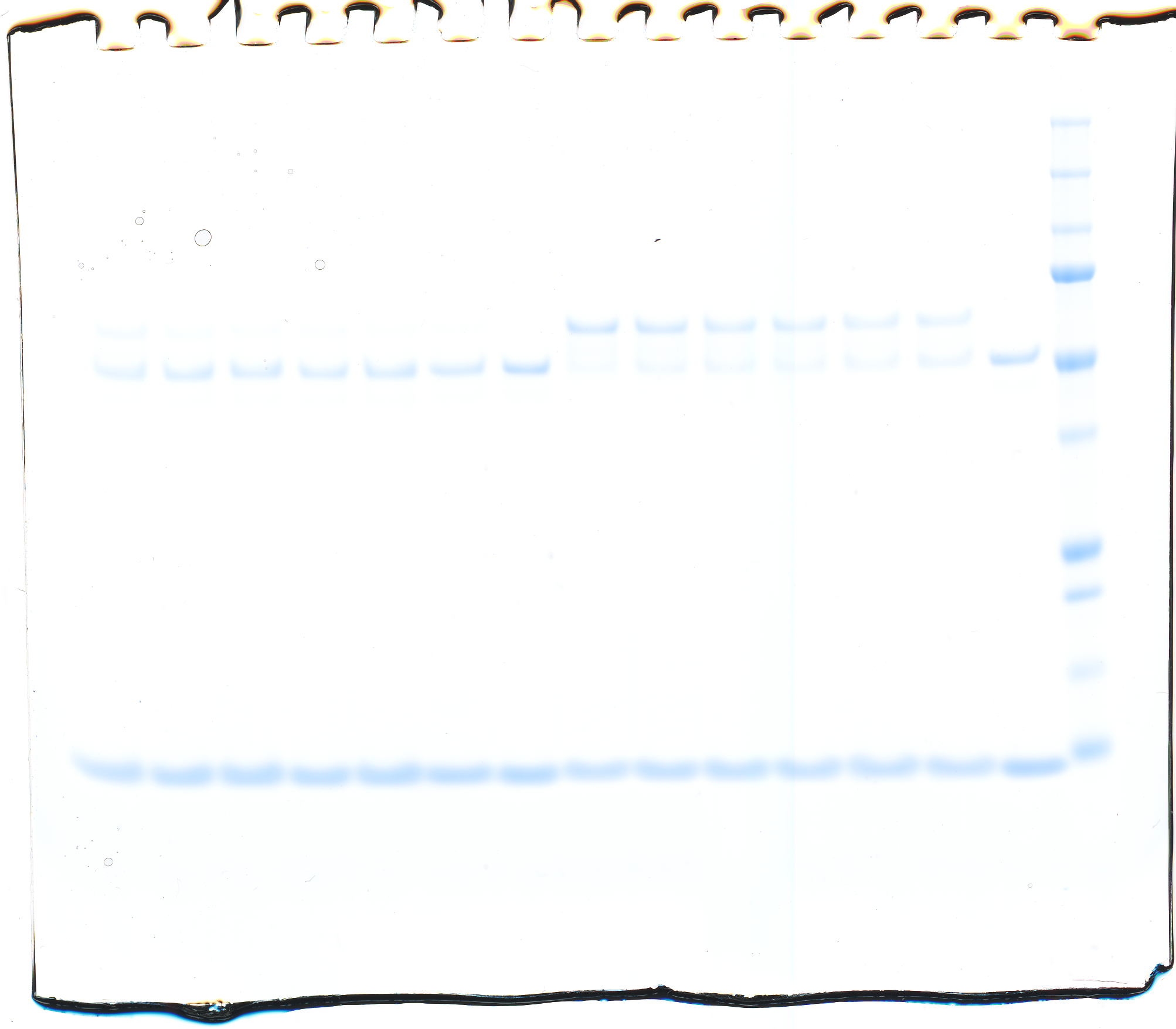

Supplement: Supplementary file 12 — Source data Fig. 8 [file 44321_2026_389_MOESM12_ESM.zip › Figure_8/8D/Figure8D.tif]

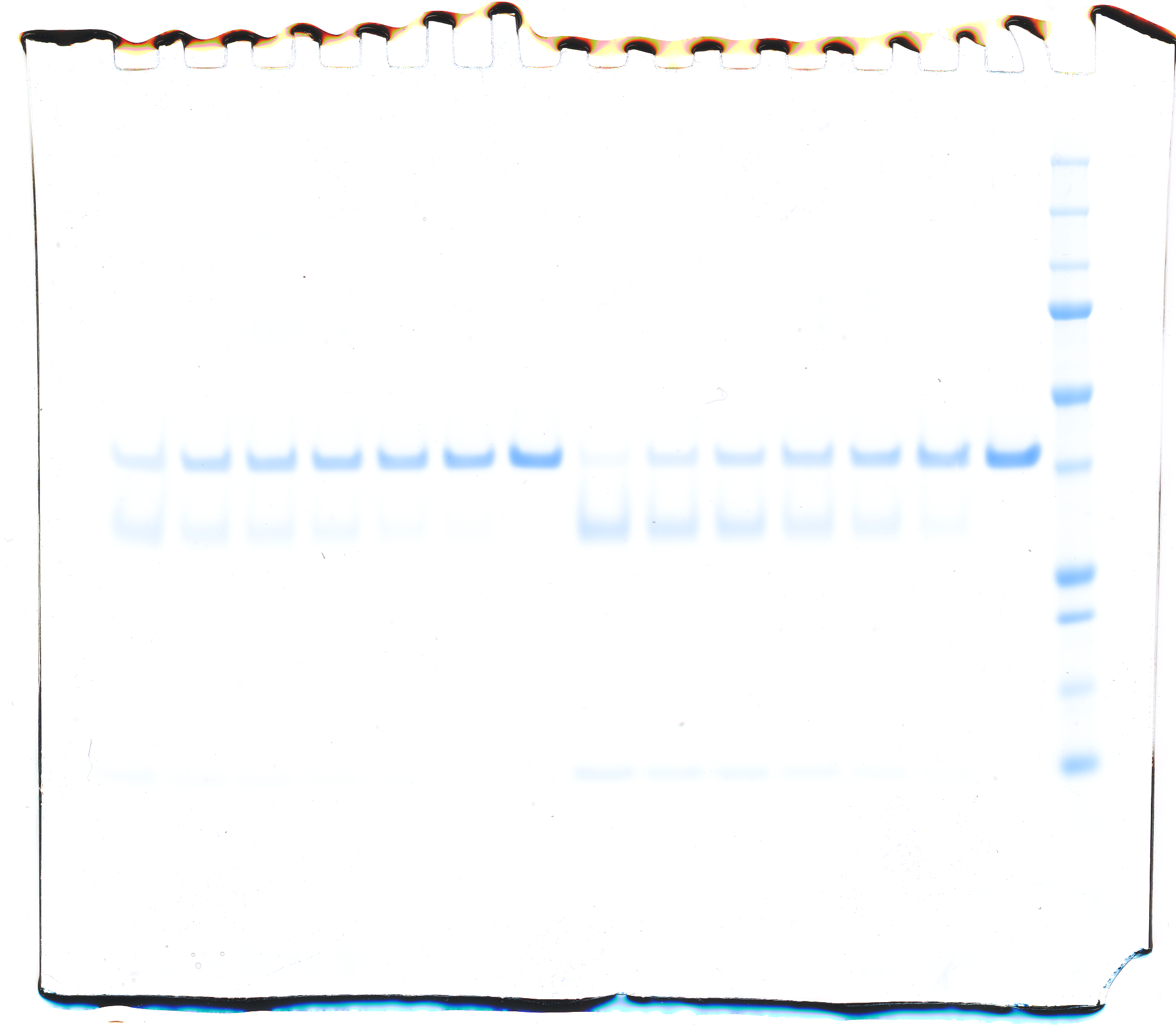

Supplement: Supplementary file 12 — Source data Fig. 8 [file 44321_2026_389_MOESM12_ESM.zip › Figure_8/8B/Figure8B.tif]

Figure 9 B

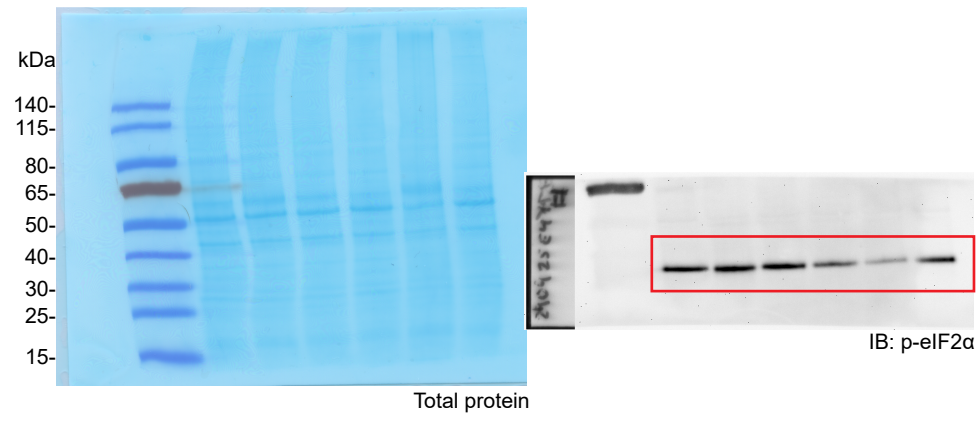

Supplement: Supplementary file 13 — Source data Fig. 9 [file 44321_2026_389_MOESM13_ESM.zip › Figure_9/9B/blot_9B.pdf]

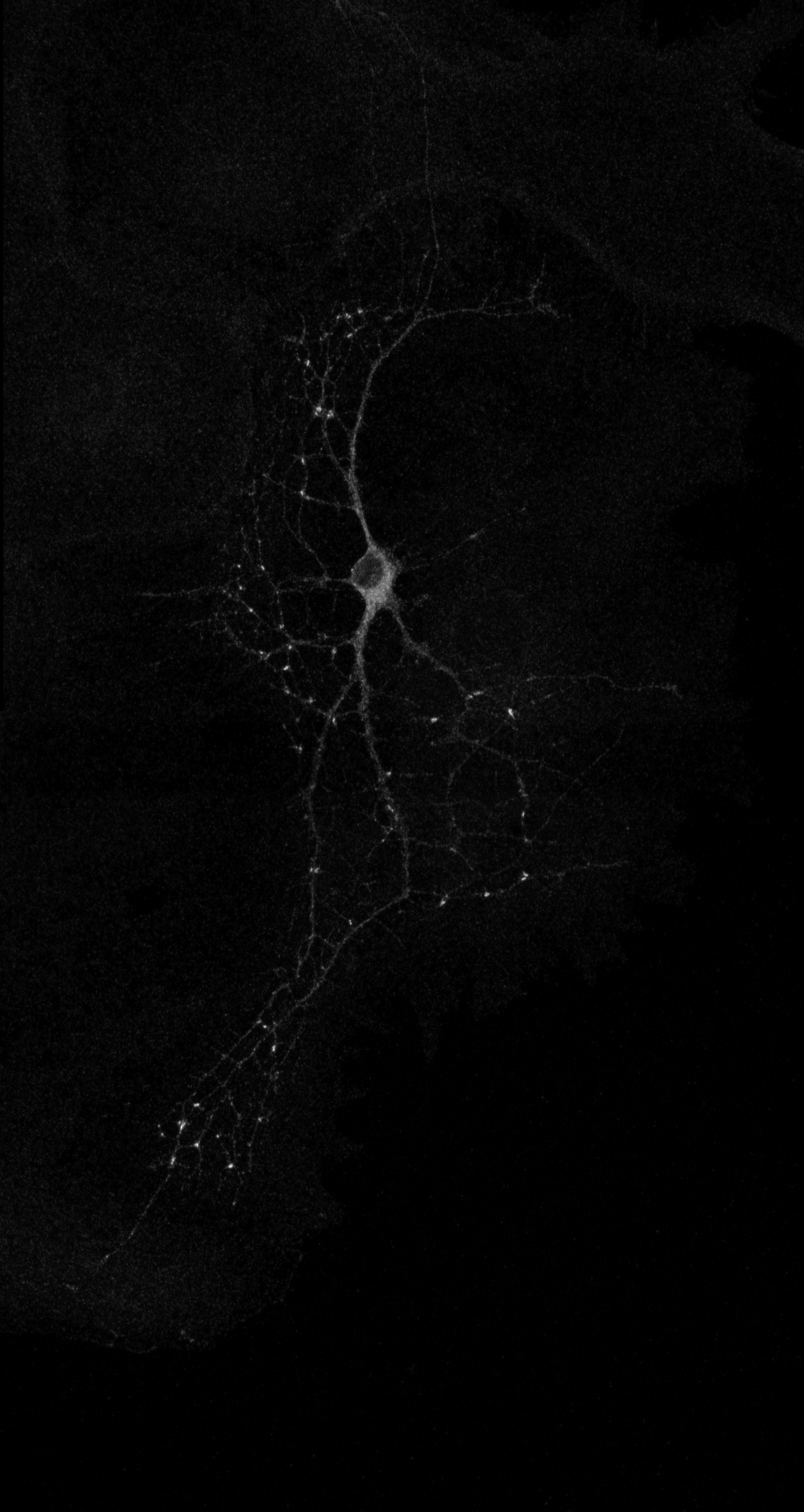

Supplement: Supplementary file 14 — Source data Fig. 10 [file 44321_2026_389_MOESM14_ESM.zip › Figure_10/10A/RFP-Control.tif]

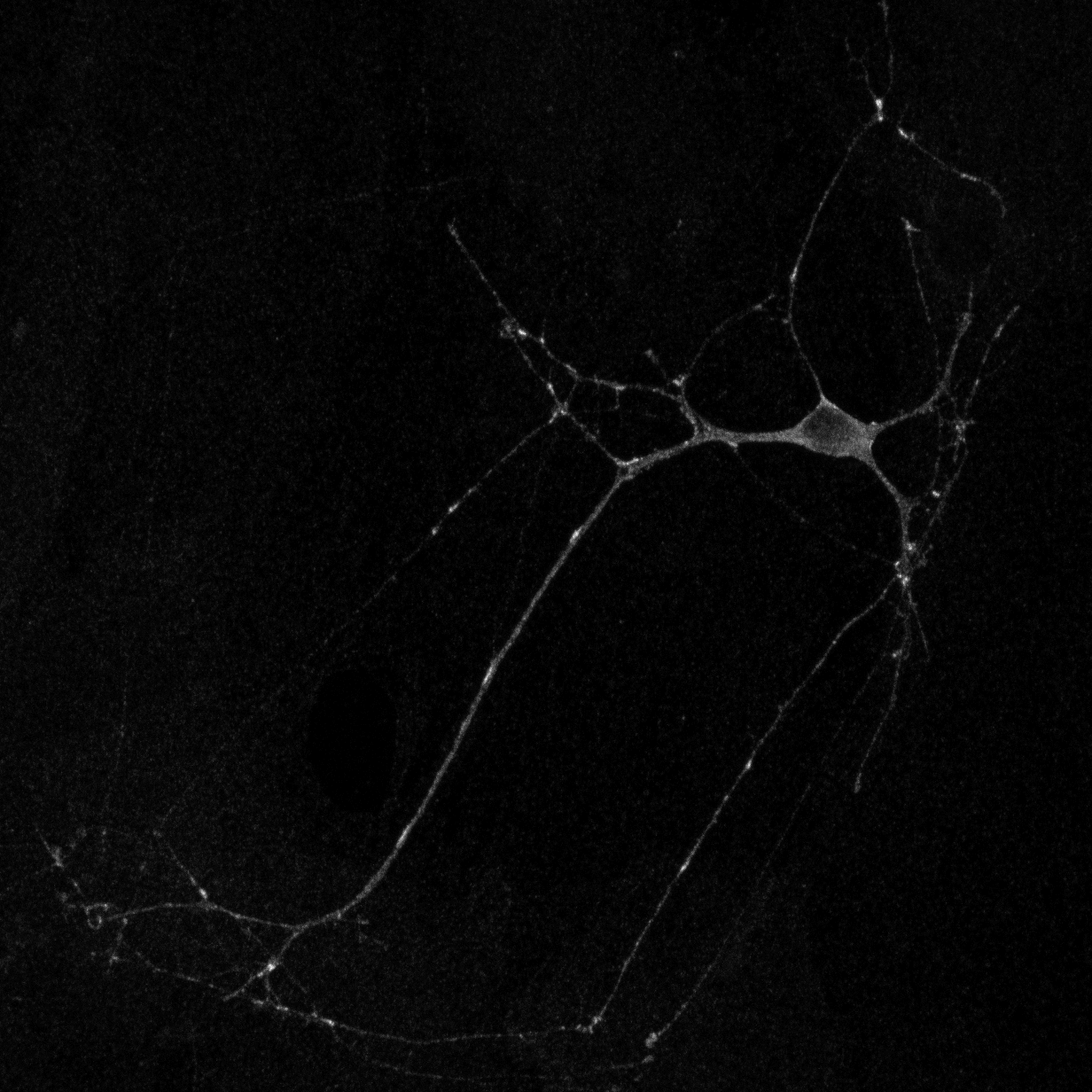

Supplement: Supplementary file 14 — Source data Fig. 10 [file 44321_2026_389_MOESM14_ESM.zip › Figure_10/10A/CRE-Control.tif]

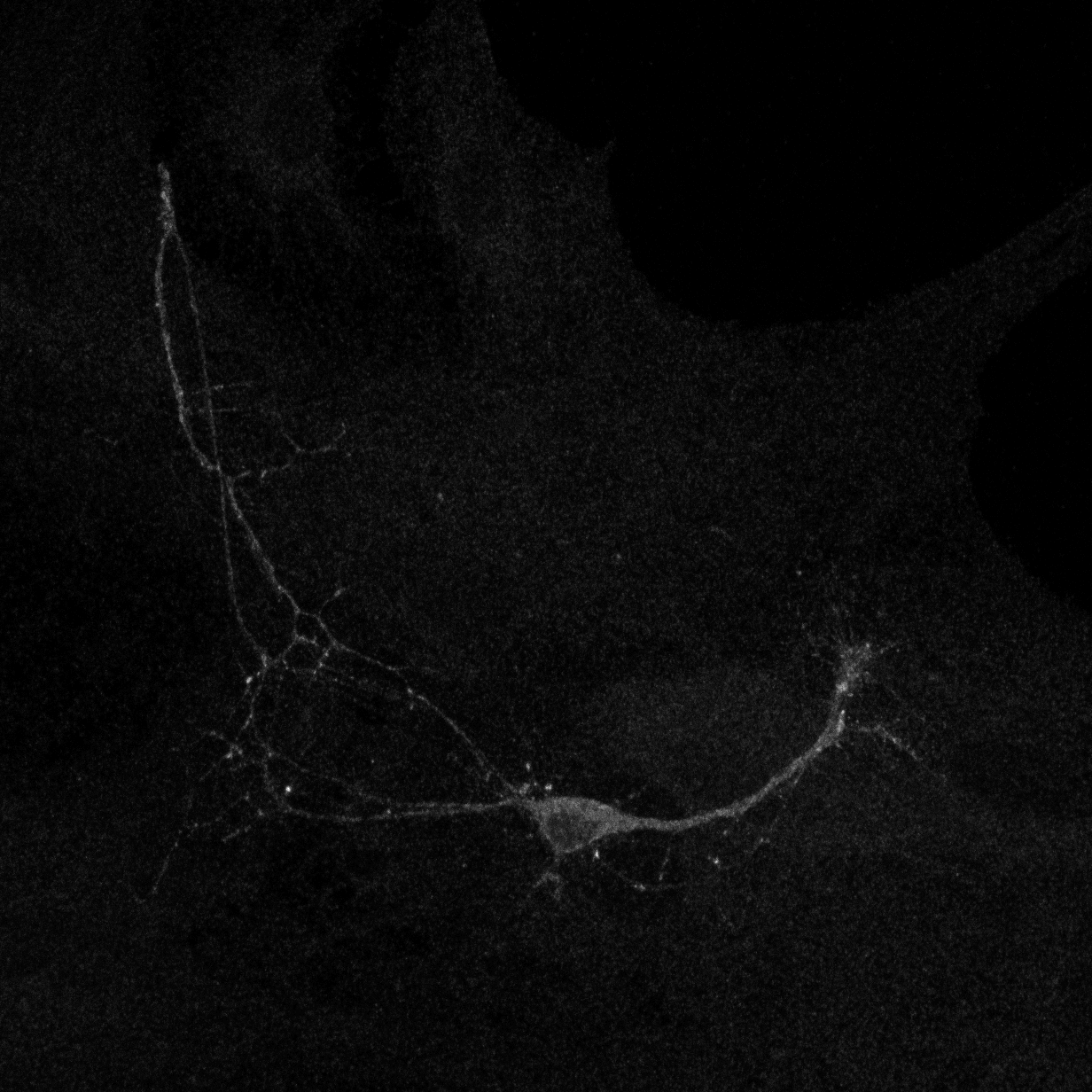

Supplement: Supplementary file 14 — Source data Fig. 10 [file 44321_2026_389_MOESM14_ESM.zip › Figure_10/10A/RC-Traz.tif]

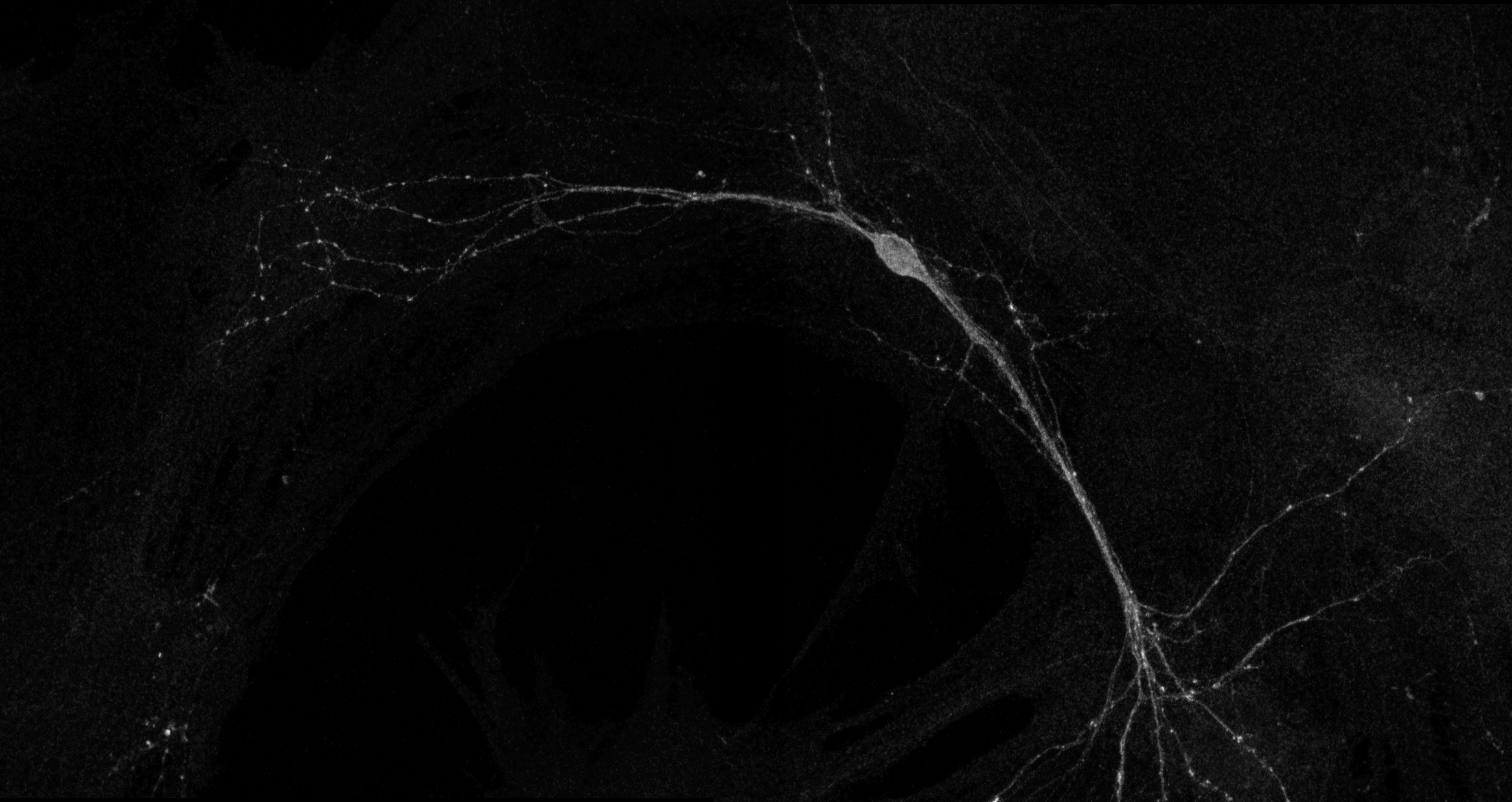

Supplement: Supplementary file 14 — Source data Fig. 10 [file 44321_2026_389_MOESM14_ESM.zip › Figure_10/10A/RRFP-Traz.tif]

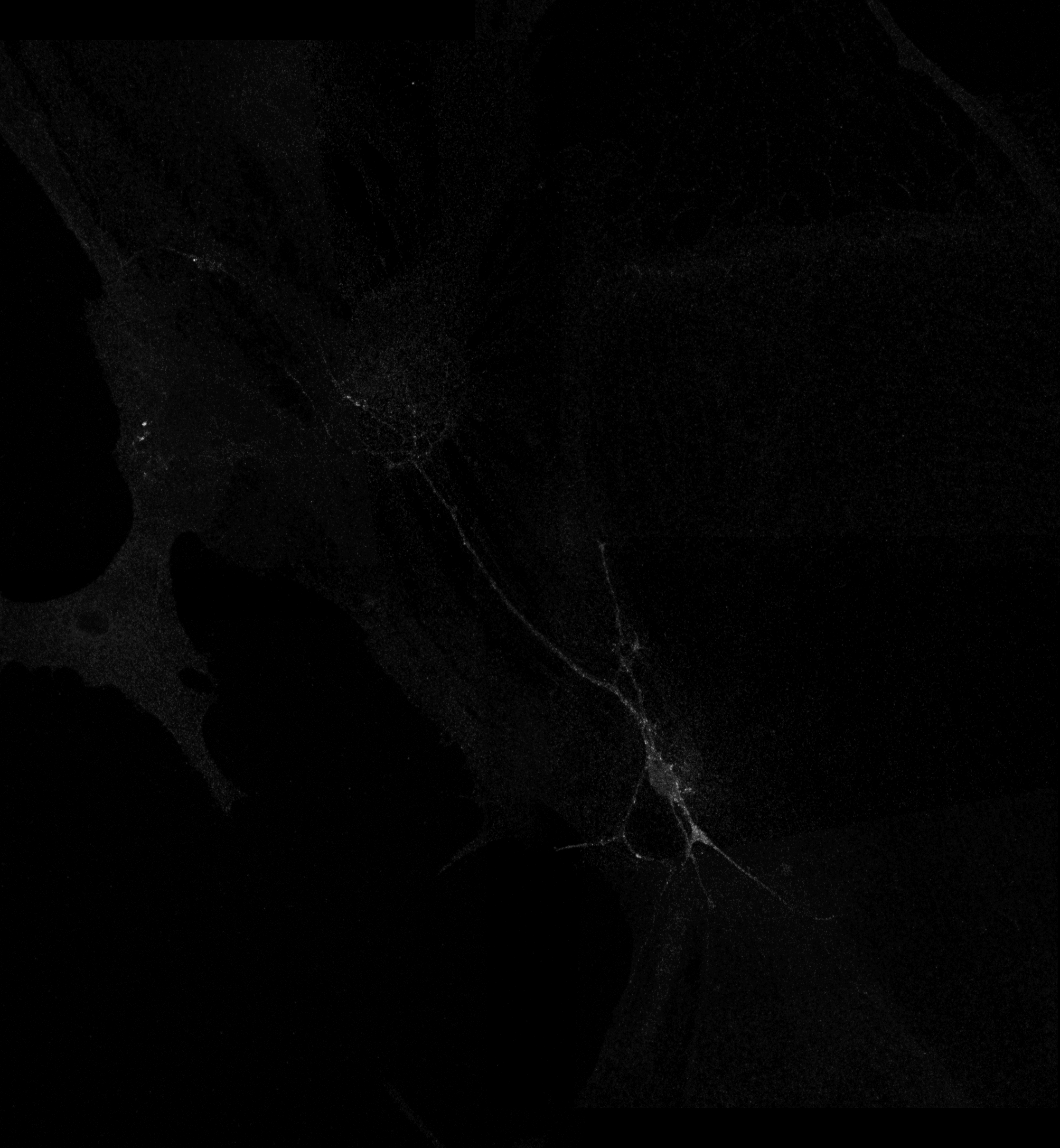

Supplement: Supplementary file 14 — Source data Fig. 10 [file 44321_2026_389_MOESM14_ESM.zip › Figure_10/10A/CRE-Traz.tif]

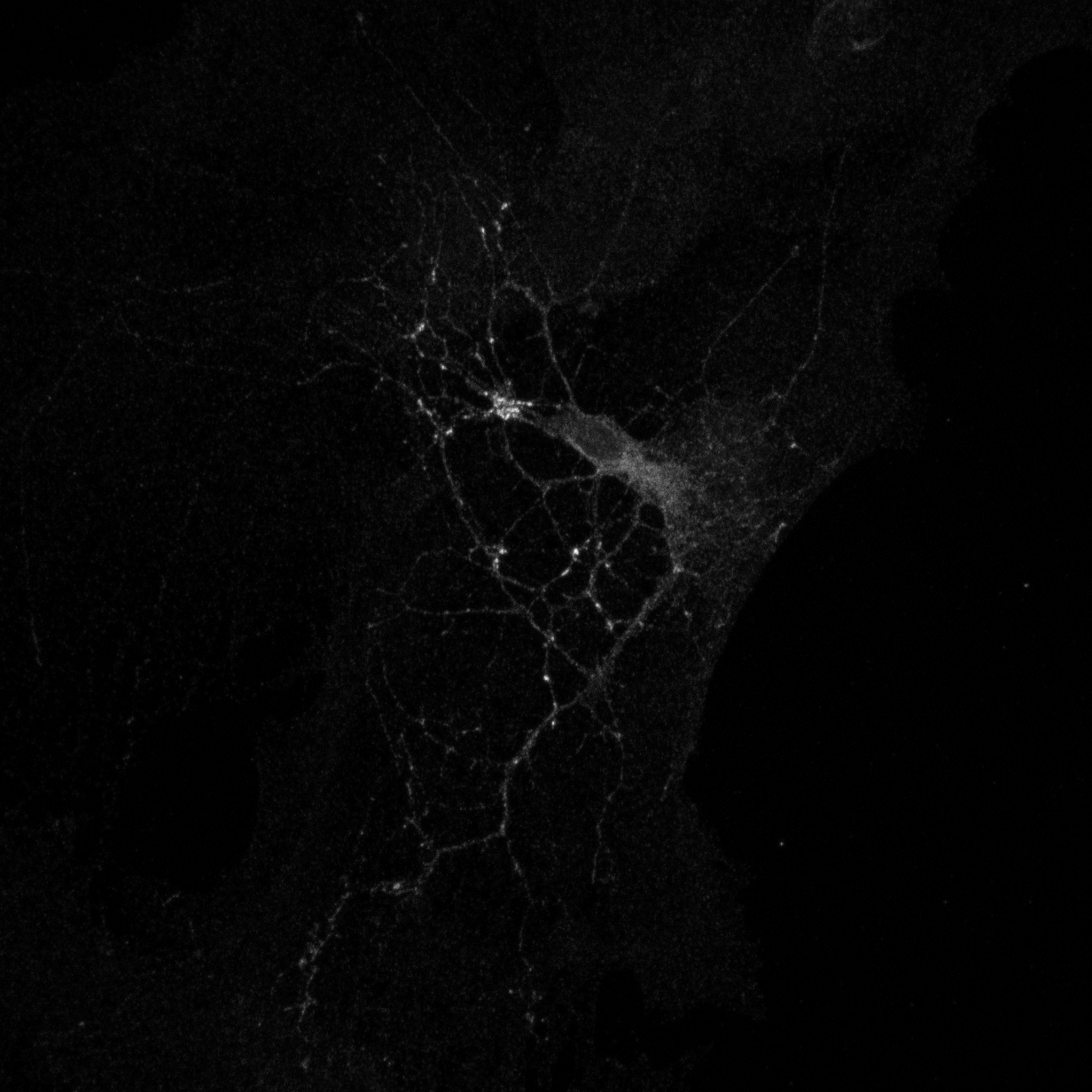

Supplement: Supplementary file 14 — Source data Fig. 10 [file 44321_2026_389_MOESM14_ESM.zip › Figure_10/10A/RC-Control.tif]
